# Electronically Driven Regioselective Iridium‐Catalyzed C−H Borylation of Donor‐π‐Acceptor Chromophores Containing Triarylboron Acceptors

**DOI:** 10.1002/chem.202002348

**Published:** 2020-07-23

**Authors:** Florian Rauch, Johannes Krebs, Julian Günther, Alexandra Friedrich, Martin Hähnel, Ivo Krummenacher, Holger Braunschweig, Maik Finze, Todd B. Marder

**Affiliations:** ^1^ Institut für Anorganische Chemie and Institute, for Sustainable Chemistry & Catalysis with Boron (ICB) Julius-Maximilians-Universität Würzburg Am Hubland 97074 Würzburg Germany

**Keywords:** boronate ester, borylation, luminescence, redox, triarylborane

## Abstract

We observed a surprisingly high electronically driven regioselectivity for the iridium‐catalyzed C−H borylation of donor‐π‐acceptor (d‐π‐A) systems with diphenylamino (**1**) or carbazolyl (**2**) moieties as the donor, bis(2,6‐bis(trifluoromethyl)phenyl)boryl (**B(^F^Xyl)_2_**) as the acceptor, and 1,4‐phenylene as the π‐bridge. Under our conditions, borylation was observed only at the sterically least encumbered *para*‐positions of the acceptor group. As boronate esters are versatile building blocks for organic synthesis (C−C coupling, functional group transformations) the C−H borylation represents a simple potential method for post‐functionalization by which electronic or other properties of d‐π‐A systems can be fine‐tuned for specific applications. The photophysical and electrochemical properties of the borylated (**1‐(Bpin)_2_**) and unborylated (**1**) diphenylamino‐substituted d‐π‐A systems were investigated. Interestingly, the borylated derivative exhibits coordination of THF to the boronate ester moieties, influencing the photophysical properties and exemplifying the non‐innocence of boronate esters.

## Graphical abstract

**That's the spot**! A surprisingly high regioselectivity was observed for an iridium‐catalyzed C−H borylation of conjugated donor‐π‐acceptor systems with bis(2,6‐bis(trifluoromethyl)phenyl)boryl (**B(^F^Xyl)_2_**) as the acceptor group. The borylated system exhibits an unexpected hypsochromic shift of the emission from toluene to THF, likely due to coordination to the boronate ester moieties (see figure).

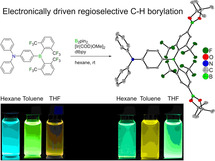

## Introduction

**Scheme 1:**
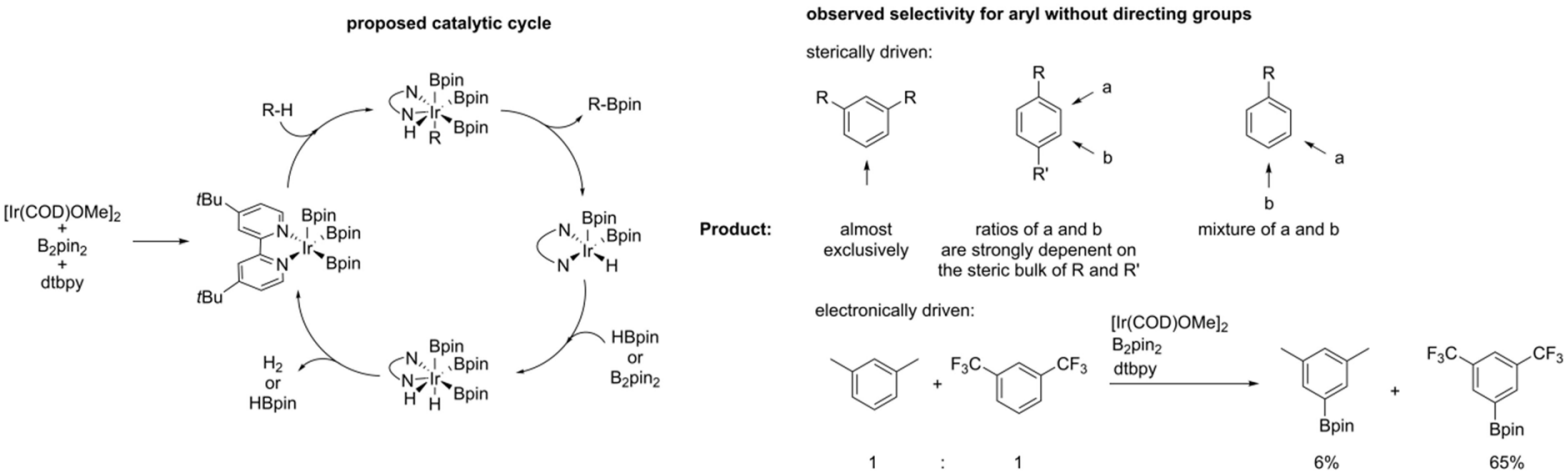
Proposed mechanism for the iridium catalyzed C−H borylation (left).^92^, ^100^ Sterically and electronically driven selectivity of the C−H borylation of arenes without directing groups (right).^89^, ^90^, ^92^, ^95^, ^100^, ^101^

In recent decades, three‐coordinate boron‐containing conjugated systems, such as triarylboranes have been of high academic interest.^1^, ^2^, ^3^, ^4^, ^5^, ^6^, ^7^, ^8^, ^9^, ^10^, ^11^, ^12^, ^13^, ^14^, ^15^ The relevance of boron in these systems is due to the empty p_z_‐orbital on boron that can act as an electron acceptor (A) in conjugated systems. Based on this, potential applications of three‐coordinate boron have been investigated, such as for linear^16^, ^17^, ^18^, ^19^, ^20^, ^21^, ^22^, ^23^, ^24^, ^25^, ^26^, ^27^, ^28^, ^29^, ^30^, ^31^, ^32^, ^33^, ^34^, ^35^, ^36^, ^37^ and non‐linear^38^, ^39^, ^40^, ^41^, ^42^, ^43^, ^44^, ^45^, ^46^, ^47^, ^48^, ^49^, ^50^ optics, bioimaging,^32^, ^49^, ^50^, ^51^, ^52^ sensors,^7^, ^53^, ^54^, ^55^ frustrated Lewis pairs (FLPs),^56^, ^57^, ^58^, ^59^, ^60^, ^61^, ^62^ and organic light‐emitting diodes (OLEDs).^63^, ^64^, ^65^ The drawback of the employment of three‐coordinate boron in conjugated systems, however, is their inherent reactivity towards nucleophiles, such as water, due to the empty p_z_‐orbital. This can be avoided by using bulky substituents or by fixing the boron center in a rigid scaffold.^25^, ^66^, ^67^, ^68^ Only recently the use of *ortho*‐trifluoromethylated aryls in triarylboranes has been established as a strategy to improve acceptor strength as well as stability.^9^, ^28^, ^30^, ^37^, ^69^, ^70^, ^71^, ^72^, ^73^, ^74^, ^75^, ^76^, ^77^, ^78^, ^79^, ^80^, ^81^ The improved acceptor strength can be attributed to the electron‐withdrawing nature of the trifluoromethyl groups, while the increase in stability is due to steric shielding of the boron center and its empty p_z_‐orbital, as well as a direct interaction of the fluorine lone pairs with the empty p_z_‐orbital on boron. The introduction of boron into organic systems can be efficiently carried out via a metalation/borylation strategy with different metal‐stabilized organic nucleophiles such as organolithium^82^, ^83^, ^84^ or Grignard reagents^68^, ^85^ with boron halides or alkoxides.^15^, ^86^ Furthermore, boron can also be introduced by transition‐metal‐catalyzed C−X^87^, ^88^ or C−H^89^, ^90^, ^91^ activation.^92^, ^93^ One of the most widely used and efficient catalytic systems is the iridium catalyzed C−H borylation using [Ir(COD)OMe]_2_ (COD=1,5‐cyclooctadiene) as the precatalytic species, bis(pinacolato)diboron (B_2_pin_2_) as the boron source and 4,4’‐di‐*tert*‐butyl‐2,2’‐bipyridine (dtbpy) as the ligand (Scheme 1).^89^, ^90^, ^93^, ^94^, ^95^, ^96^, ^97^, ^98^, ^99^

This catalytic system has been thoroughly investigated and exhibits a high regioselectivity due to the sterically bulky nature of the proposed catalytically active species.^31^, ^90^, ^95^, ^102^, ^103^, ^104^, ^105^, ^106^, ^107^, ^108^, ^109^, ^110^, ^111^ Additionally, the regioselectivity can also be influenced by functional groups^96^, ^97^, ^99^, ^112^, ^113^ or by modification of the ligand.^114^, ^115^, ^116^, ^117^ The importance of the iridium‐catalyzed C−H borylation is due to the use of boronate esters and boronic acids in Suzuki–Miyaura cross‐coupling^118^, ^119^, ^120^, ^121^ as well as the possibility of functional‐group transformations.^86^, ^92^, ^101^, ^118^, ^122^, ^123^, ^124^, ^125^, ^126^, ^127^, ^128^ In addition to the steric influence on the regioselectivity, there is also an electronic component.^129^ A high degree of electronically directed regioselectivity was observed for the borylation of quinolines^130^ and the borylation of hetero‐aryls.^131^, ^132^, ^133^, ^134^

We have previously investigated donor‐π‐acceptor (d‐π‐A) systems with *bis*(2,6‐*bis*(trifluoromethyl)phenyl)boryl (**B(^F^Xyl)_2_**) as the acceptor group.^37^ To fine tune the acceptor properties we wanted to look for a regioselective methodology to functionalize the acceptor in d‐π‐A systems, exclusively. Based on previous results, we decided to use the iridium‐catalyzed C−H borylation and found it to exhibit a surprisingly high electronically driven regioselectivity for our systems.

## Results and Discussion

### Synthesis

**Scheme 2:**
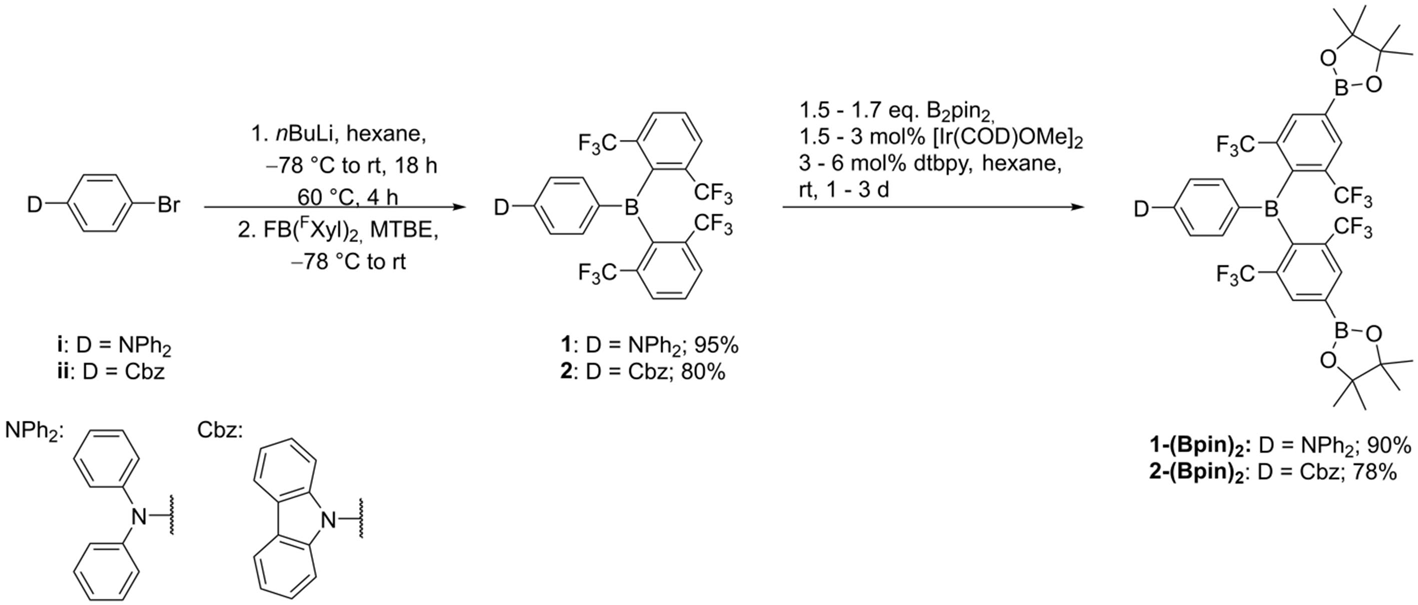
Synthesis of **1**, **2**,^37^ **1‐(Bpin)_2_**, and **2‐(Bpin)_2_**.

Compound **1** was synthesized analogously to our previously published methodology for donor‐π‐acceptor (d‐π‐A) compound **2** (Scheme 2).^37^ The brominated donor‐bridge moiety (**i** and **ii**) was lithiated using *n*BuLi in hexane. Afterwards, bis(2,6‐bis(trifluoromethyl)phenyl)fluoroborane (**FB(^F^Xyl)_2_**) was added in methyl *tert*‐butyl ether (MTBE). We found that the solvent mixture is important. When Et_2_O is used for the lithiation, the lithiation is much faster, but the reaction with **FB(^F^Xyl)_2_** is strongly inhibited. The amount of coordinating solvents, even weakly coordinating ones, has a strong influence on the yield and duration of the reaction. We assume that the solvent coordinates to the fluoroborane, thereby reducing the reactivity towards nucleophiles. After purification via column chromatography, compounds **1** and **2** 37 were isolated in excellent yields. The iridium‐catalyzed C−H borylation was conducted in hexane at room temperature with 1.5 to 1.7 equivalents of B_2_pin_2_ and a catalyst loading of 1.5 to 3 mol % and 3 to 6 mol % ligand. The mixture of catalyst, B_2_pin_2_, and ligand was stirred in hexane for 10 min before adding the d‐π‐A compounds **1** and **2**, in order to form the catalytically active species before adding the substrate. The reaction was closely monitored via ^1^H NMR spectroscopy, and only **1‐(Bpin)**/**2‐(Bpin)**, **1‐(Bpin)_2_**/**2‐(Bpin)_2_** or unreacted **1**/**2** were detected. This high degree of regioselectivity was surprising to us, as no borylation at all at the sterically unencumbered arylamino moieties was observed. Note that the selectivity is clearly electronic in nature, as the two arenes have similar steric properties, at least at their *para*‐positions. It should be noted that, in a competitive borylation experiment of a 1:1 mixture of *m*‐xylene and 1,3‐bis(trifluoromethyl)benzene, 6 % borylation of *m*‐xylene and 65 % borylation of 1,3‐bis(trifluoromethyl)benzene were observed,^100^ illustrating the electronic preference for the borylation of electron deficient arenes or more acidic C−H bonds, as also noted by Steel and Marder.^130^ So, while selectivities of up to approximately 10:1 had been noted previously for the C−H borylation of aromatics, the selectivity in our current case seems to be significantly greater.

However, when the amount of B_2_pin_2_ is increased to 2.2 equivalents, borylation at the donor moieties can be observed via ^1^H NMR spectroscopy. Residual, small amounts of mono‐borylated d‐π‐A can be removed by washing with methanol and recrystallization. No column chromatography was needed, which is beneficial, as aryl‐boronates can be very difficult to purify by column chromatography due to their strong interaction with the solid phase. All compounds exhibit ^1^H, ^11^B, ^19^F and ^13^C NMR spectra in accordance with their structures. The ^19^F NMR spectra exhibit strong signal broadening, indicative of a hindered rotation about the B−C bonds, as previously observed for similar boranes with *ortho*‐trifluoromethyl groups.^37^, ^73^, ^81^

### Crystal and molecular structures

**Figure 1:**
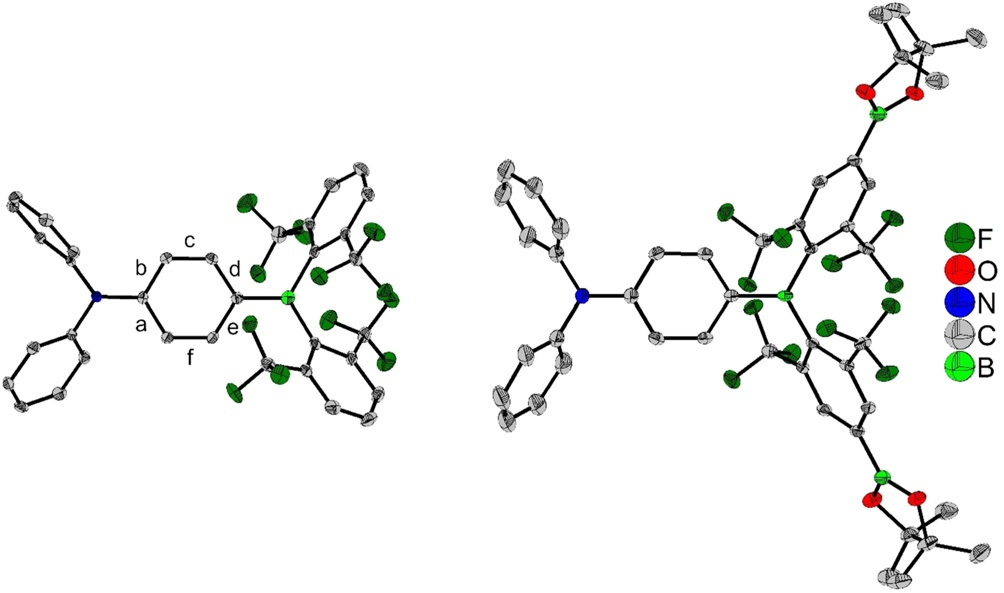
The solid‐state molecular structures of **1** (left) and **1‐(Bpin)_2_** (right) determined by single‐crystal X‐ray diffraction at 100 K. All ellipsoids are drawn at the 50 % probability level. H atoms and solvent molecules are omitted for clarity. For **1‐(Bpin)_2_**, the diphenylamino group (NPh_2_) is disordered by twofold rotational symmetry and only one part (50 %) is shown here.

Crystals suitable for X‐ray diffraction analysis of **1** and **1‐(Bpin)_2_** were obtained from a saturated hexane solution or from a saturated CH_2_Cl_2_ solution that was layered with hexane, respectively. The molecular structures are depicted in Figure 1 and selected bond lengths, distances and angles are listed in Table 1. The molecular structure of **2‐(Bpin)_2_** was also obtained via single‐crystal X‐ray diffraction (Figure S17, Supporting Information). Although the quality of the data does not allow a detailed discussion of the structural parameters, it does provide proof of the connectivity, confirming that borylation took place at the electron‐poor arene rings.

**Table 1:** Selected bond lengths, distances (Å) and angles (°) of **1** and **1‐(Bpin)_2_**.

	**1**	**1‐(Bpin)_2_**
B−C (triarylborane, internal)	1.525(3)	1.531(5)
B−C (triarylborane, terminal)	1.611(3) 1.617(3)	1.606(3) 2×
C−N (internal)	1.395(3)	1.395(4)
C−N (terminal)	1.433(3) 1.440(3)	1.436(12) 1.430(12)
Sum ∢ CBC	360.0(2)	360.00(14)
Sum ∢ CNC	358.50(17)	359.1(7)
C−C (phenylene bridge): a b c d e f	1.405(3) 1.402(3) 1.373(3) 1.414(3) 1.408(3) 1.374(3)	1.407(3) =a 1.374(3) 1.408(3) =d =c
∢BC_3_‐NC_3_	37.27(9)	15.2(2)
∢BC_3_‐phenylene (central)	20.55(18)	23.31(11)
∢BC_3_‐ ^F^Xyl (terminal) ∢BC_3_‐ ^F^Xyl (terminal)	53.51(9) 52.78(8)	51.45(7) 2×
∢NC_3_‐phenylene (central)	16.71(8)	8.2(2)
∢NC_3_‐phenyl (terminal) ∢NC_3_‐phenyl (terminal)	41.86(9) 60.10(8)	69.9(3) 71.3(3)
Shortest B⋅⋅⋅F contacts	2.769(3) 2.797(3) 2.861(3) 2.876(3)	2.822(2) 2.855(3)

The triarylboron and ‐nitrogen centers in **1** and **1‐(Bpin)_2_** exhibit trigonal planar geometries with the sum of the angles around the centers of ≈360°. Compounds **1** and **1‐(Bpin)_2_** exhibit two longer B−C bonds (1.606(3)–1.617(3) Å) towards the terminal ^F^Xyl moieties and one shorter B−C bond (1.525(3) and 1.531(3) Å) towards the phenylene bridge. This can be mainly attributed to the steric demand of the ^F^Xyl *ortho* trifluoromethyl groups. Due to the steric hinderance the ^F^Xyl moieties also exhibit larger torsion angles (51.45(7)–53.51(9)°) with respect to the BC_3_ plane than the phenylene bridge (20.55(18) and 23.31(11)°). The C−N bond lengths of **1** and **1‐(Bpin)_2_** show a similar behavior and exhibit two longer bonds (1.430(12)–1.440(3) Å) to the terminal phenyl moieties and one shorter bond (1.395(3) and 1.395(4) Å, respectively) to the phenylene bridge. As the steric demand of all three substituents at the N atom is equal, this can be attributed to a polarized ground state, which is well known for d‐π‐A systems.^49^, ^81^ This is further supported by the larger torsion angles of the terminal phenyl groups with respect to the NC_3_ plane (41.86(9)–71.3(3)°), compared to those of the phenylene bridge (16.71(8) and 8.2(2)°). The polarized ground state also results in a quinoidal distortion of the phenylene spacer, as the c and f bonds in **1** and **1‐(Bpin)_2_** (Figure 1, Table 1) (1.373(3)–1.374(3) Å) are significantly shorter than the a, b, d, and e bonds (1.402(3)–1.414(3) Å). Similar to previously reported triarylboranes bearing *ortho*‐trifluoromethyl groups,^28^, ^69^, ^73^, ^74^ the molecular structures of **1** and **1‐(Bpin)_2_** show B⋅⋅⋅F contacts (2.769(3)–2.876(3) Å), which are shorter than the sum of their van der Waals radii (3.39 Å),^135^ indicating a stabilizing interaction between the lone pairs of the CF_3_ fluorine atoms and the empty p_z_‐orbital of the boron center.

### Electrochemistry

**Figure 2:**
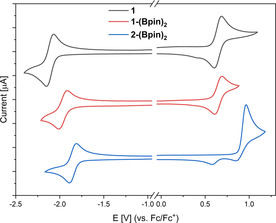
Cyclic voltammograms of **1**, **1‐(Bpin)_2_**, and **2‐(Bpin)_2_** in CH_2_Cl_2_. All samples are referenced to the Fc/Fc^+^ redox couple.

Cyclic voltammograms of **1**, **1‐(Bpin)_2_**, and **2‐(Bpin)_2_** were recorded in CH_2_Cl_2_ with [*n*Bu_4_N][PF_6_] as the electrolyte and a scan rate of 250 mVs^−1^ (Figure 2) in order to determine their reduction and oxidation potentials, which are referenced to the ferrocene/ferrocenium redox couple (Fc/Fc^+^) and listed in Table 2. For comparison, our previously reported d‐π‐A systems **2**,^37^ 4‐(dimesitylboryl)‐*N*,*N*‐diphenylaniline (**I**),^73^ and 4‐(bis(2,4,6‐tris(trifluoromethyl)phenyl)boryl)‐*N*,*N*‐diphenylaniline (**II**)^73^ are also listed in Table 2.

**Table 2:** Reduction potentials of **1**, **1‐(Bpin)_2_**, **2**, **2‐(Bpin)_2_**, **I**, and **II** referenced to the Fc/Fc^+^ redox couple.

	*E*_*1/2*_ vs. Fc/Fc^+^ [V]	
Compound	red	ox
**1**	−2.11	0.64
**1‐(Bpin)_2_**	−1.97	0.65
**2** 37	−1.96	0.96 (irr)^[a]^
**2‐(Bpin)_2_**	−1.86	0.96 (irr)^[a]^
**I** 73	−2.60	0.39
**II** 73	−1.66	0.72

[a] For irreversible oxidation events *E*_p.a_ is given.

Compounds **1** (*E*_1/2, red_=−2.11 V; *E*_1/2 ox_=0.64 V) and **1‐(Bpin)_2_** (*E*_1/2, red_=−1.97 V; *E*_1/2, ox_=0.65 V) both exhibit reversible reduction and oxidation waves, that can be attributed to the triarylboron and nitrogen centers, respectively. **2‐(Bpin)_2_** (*E*_1/2, red_=−1.86 V; *E*_p.a, ox_=0.96 V) exhibits a reversible reduction corresponding to the triarylboron center and an irreversible oxidation characteristic of the carbazolyl moiety, analogous to what was previously observed for **2** (*E*_1/2, red_=−1.96 V; *E*_p.a., ox_=0.96 V).^37^ The reduction potential of **1** is 150 mV cathodically shifted compared to **2**, indicating that diphenylamine is a stronger donor than carbazolyl.^136^, ^137^ The introduction of a Bpin group *para* to the boron on the ^F^Xyl moieties shifts the reduction potentials anodically by 140 and 100 mV, respectively for **1** and **2**. This indicates the electron‐withdrawing nature of the boronate ester moieties. This is in line with our previously published comprehensive comparison of different boron based acceptor groups.^138^ The reduction potential of **1** is anodically shifted by 490 mV compared to its mesityl analogue (**I**), and cathodically shifted by 450 mV compared to its fluoromesityl analogue (**II**).^73^ This indicates the stronger electron‐withdrawing effect of the trifluoromethyl group *para* to the boron center as compared to the *ortho* trifluoromethyl groups.^139^

### Photophysical properties

**Figure 3:**
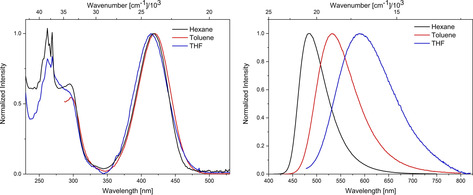
Normalized absorption (left) and emission (right) spectra of **1** in hexane (black), toluene (red), and THF (blue).

**Figure 4:**
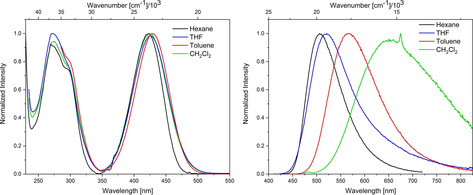
Normalized absorption (left) and emission (right) spectra of **1‐(Bpin)_2_** in hexane (black), toluene (red), THF (blue) and CH_2_Cl_2_ (green).

The photophysical properties of **1** (Figure 3, Table 3) and **1‐(Bpin)_2_** (Figure 4, Table 3) were examined in solvents of increasing polarity. Additionally, the photophysical properties of **2‐(Bpin)_2_** were also examined in hexane (Figure S18, Table 3).

**Table 3:** Photophysical properties of **1**, **1‐(Bpin)_2_**, and **2‐(Bpin)_2_**.

Compd.	Solvent	*λ*_max_ (abs.) [nm]	*ϵ* [M^−1^ cm^−1^]	*λ*_max_ (em.) [nm]	Apparent Stokes Shift [cm^−1^]	*τ*_f_ [ns]	*τ*_0_ [ns]^[a]^	*Φ*_f_
**1**	hexane	418	35 000	484	3400	10.3	10.5	0.98
	toluene	420		532	5000	9.9	11.1	0.89
	THF	414		590	7200	2.4^[b]^	30.0	0.08
**1‐(Bpin)_2_**	hexane	423	13 000	506	3900	8.8	13.8	0.64
	toluene	428		566	5700	4.7	18.8	0.25
	THF	426		520	4200	7.2	36.0	0.20
	CH_2_Cl_2_	424		650	8200	N.D.	N.D.	N.D.
**2‐(Bpin)_2_**	hexane	398		433	2000	8.9	10.0	0.89

[a] Calculated from *τ*_f_/*Φ*_f_. [b] Calculated average of two lifetimes: 1.7 ns (90 %), 8.6 ns (10 %).

Compound **1** exhibits a broad, structureless lowest energy absorption at ***λ***_max, abs_=418, 420, and 414 nm in hexane, toluene and THF, respectively. No shift of the absorption spectra as a function of solvent polarity is observed, indicating only a weakly polarized ground state. TD‐DFT calculations on **1** at the CAM‐B3LYP/6–31+G(d) level of theory suggest that the S_1_←S_0_ transition can be attributed to a HOMO to LUMO transition. The HOMO is localized on the donor and π‐bridge, and the LUMO is mainly localized on the acceptor. As such, the transition can be classified as a charge transfer (CT) transition. Similar observations were made for our previously reported d‐π‐A systems with B(^F^Xyl)_2_, B(^F^Mes)_2_ or BMes_2_ as the acceptor.^30^, ^37^, ^73^, ^140^ In accordance with a CT transition, the emission maximum of **1** shifts bathochromically with increasing solvent polarity (***λ***_max, em_=484, 532, and 590 nm in hexane, toluene and THF, respectively). This is expected for dipolar d‐π‐A systems, due to the stabilization of the excited state in solvents of higher polarity. In comparison to our previously investigated analogues of **1** with BMes_2_ (**I**) and B(^F^Mes)_2_ (**II**), both absorption and emission are bathochromically shifted compared to **I** (***λ***_max, abs_=377, 380, 378 nm; ***λ***_max, em_=410, 437, 462 nm in hexane, toluene and THF, respectively) and hypsochromically shifted compared to **II** (***λ***_max, abs_=444, 448, 441 nm; ***λ***_max, em_=563, 638, 743 nm in hexane, toluene and THF, respectively). This supports the results from the cyclovoltammetry measurements, in that B(^F^Xyl)_2_ is right in the middle between BMes_2_ and B(^F^Mes)_2_ in terms of acceptor strength. The lowest energy absorption maxima of **1‐(Bpin)_2_** are slightly red shifted compared to **1** (***λ***_max, abs_=423, 428, 426, and 424 nm in hexane, toluene, THF and CH_2_Cl_2_, respectively). The emission maxima of **1‐(Bpin)_2_** in hexane (***λ***_max, em_=506 nm) and toluene (***λ***_max, em_=566 nm) are also red‐shifted compared to **1** and also exhibit a bathochromic shift with increasing solvent polarity. In THF, however, the emission maximum (***λ***_max, em_=520 nm) of **1‐(Bpin)_2_** is blue shifted compared to those of **1** and **1‐(Bpin)_2_** in toluene. This is unexpected, as due to the higher polarity of THF a further bathochromic shift is to be expected. We suspect, that this is due to coordination of THF to the boron centers of the boronate ester moieties, as a result of which the Bpin moieties change from being weak acceptors to weak donors, decreasing the acceptor strength of the B(^F^Xyl)_2_ moiety, resulting in a hypsochromic shift. This is further supported by the strongly bathochromically shifted and very weak emission of **1‐Bpin_2_** in CH_2_Cl_2_, a non‐coordinating polar solvent. Hence, the lowest energy transitions of **1‐Bpin_2_** can also be classified as CT transitions. Compared to **1‐(Bpin)_2_**, **2‐(Bpin)_2_** exhibits blue‐shifted lowest energy absorption (***λ***_max, abs_=398 nm) and emission (***λ***_max, em_=433 nm) maxima in hexane. This can be attributed to carbazolyl being a weaker donor than diphenylamine, resulting in a weaker charge transfer (CT).^136^, ^137^ Similarly, the previously reported lowest energy absorption (***λ***_max, abs_=400, 398, and 387 nm) and emission (***λ***_max, em_=423, 470, and 524 nm) maxima of **2** 37 are also blue shifted compared to those of **1** in hexane, toluene, and THF, respectively. The quantum yield of **1** in hexane is close to unity, which is similar to that previously observed for **2**. Similar to **2**, the quantum yield of **1** in toluene is still high, but unlike **2**, the quantum yield of **1** drops off drastically in THF. This could be due to the energy gap law, as internal conversion processes become more effective, as the energy gap between the excited and ground state becomes smaller, which we previously observed for similar systems.^30^, ^73^ The quantum yields of **1‐(Bpin)_2_** and **2‐(Bpin)_2_** in hexane are lower than those of their respective unborylated parent compounds **1** and **2**. The same was observed for **1‐(Bpin)_2_** in toluene. Interestingly, the quantum yield of **1‐(Bpin)_2_** is higher in THF than that of **1**. This is likely a result of the blue‐shifted absorption and emission maxima of **1‐(Bpin)_2_** as compared with **1**.

## Conclusions

While looking for a methodology to post‐functionalize d‐π‐A systems, we found a surprisingly high electronically driven regioselectivity for an iridium‐catalyzed C−H borylation using [Ir(COD)OMe]_2_ as the precatalytic species, B_2_pin_2_ as the boron source and dtbpy as the ligand in hexane.^89^, ^90^, ^93^, ^94^, ^95^, ^96^, ^97^, ^98^, ^99^ We believe that, due to the versatility of boronate esters for C−C coupling or functional‐group transformations, this provides a good handle for post‐functionalization of d‐π‐A systems in order to fine tune the electronic properties as suggested by the literature.^86^, ^92^, ^101^, ^118^, ^119^, ^120^, ^121^, ^122^, ^123^, ^124^, ^125^, ^126^, ^127^, ^128^ This is especially useful in light of our recent paper on the application of triarylboranes as acceptors in compounds exhibiting thermally activated delayed fluorescence, that illustrates the importance of the relative energy levels of donor, π‐bridge and acceptor moiety.^37^ The investigation of the electrochemical properties shows that B(^F^Xyl)_2_ is a stronger acceptor than BMes_2_ and a weaker acceptor than B(^F^Mes)_2_, and provides the possibility of further functionalization due to the unsubstituted *para* position. This is also supported by the photophysical data of **1**, **1‐(Bpin)_2_**, and **2‐(Bpin)_2_**. The borylated derivatives are slightly red shifted compared to their parent compounds, indicating an electron‐withdrawing effect of the Bpin moieties. The compounds show CT transitions with bathochromic shifts of the emission with increasing solvent polarity. A surprising exception is **1‐(Bpin)_2_**, which exhibits a hypsochromic shift of the emission when increasing the solvent polarity from toluene to THF. This is attributed to a coordination of THF to the boron centers of the boronate ester groups and a subsequent weakening of its acceptor strength.

## Conflict of interest

The authors declare no conflict of interest.

## Supporting information

As a service to our authors and readers, this journal provides supporting information supplied by the authors. Such materials are peer reviewed and may be re‐organized for online delivery, but are not copy‐edited or typeset. Technical support issues arising from supporting information (other than missing files) should be addressed to the authors.

Supplementary
