# Supplementary material for: Electronically Driven Regioselective Iridium‐Catalyzed C−H Borylation of Donor‐π‐Acceptor Chromophores Containing Triarylboron Acceptors
Source: Chemistry. 2020 Jul 23;26(46):10626–33. doi: 10.1002/chem.202002348 (PMC7497074; doi:10.1002/chem.202002348)
Supplement: Supplementary file 1 — Supplementary [file CHEM-26-10626-s001.pdf]

Chemistry–A European Journal

Supporting Information

Electronically Driven Regioselective Iridium-Catalyzed C−H Borylation of Donor-π-Acceptor Chromophores Containing Triarylboron Acceptors

Florian Rauch, Johannes Krebs, Julian Günther, Alexandra Friedrich, Martin Hähnel, Ivo Krummenacher, Holger Braunschweig, Maik Finze, and Todd B. Marder*[a]

Supporting Information

Table of Contents

General experimental details 2
Synthesis 5
4-(Bis(2,6-bis(trifluoromethyl)phenyl)boryl)-*N*,*N*-diphenylaniline (1) 5
4-(Bis(4-(4,4,5,5-tetramethyl-1,3,2-dioxaborolan-2-yl)-2,6-bis(trifluoromethyl)phenyl)boryl)-N,N-diphenylaniline (1-(Bpin)$_2$) 6
9-(4-(Bis(4-(4,4,5,5-tetramethyl-1,3,2-dioxaborolan-2-yl)-2,6-bis(trifluoromethyl)phenyl)boryl)phenyl)-9H-carbazole (2-(Bpin)$_2$) 7
NMR Spectra 8
Crystal structure determinations 15
Photophysical data 19
Electrochemistry 20
TD-DFT Calculations 22
Theoretical calculations: Cartesian coordinates 24
References 25

General experimental details

Unless otherwise noted, the following conditions apply.

All syntheses were carried out using standard Schlenk and glovebox techniques under an argon atmosphere. The solvents used were dried using a solvent purification system (SPS) from Innovative Technology and were degassed and stored over molecular sieves under argon. Deuterated solvents (CD_2Cl_2, $CDCl_3$, C_6D_6) used for NMR spectroscopy were purchased from Cambridge Isotope Laboratories. C_6D_6 was dried over molecular sieves and stored under an argon atmosphere before use. Boron trifluoride diethyl etherate was purchased from Merck Millipore. *n*-Butyllithium (2.5 M solution in hexane) was purchased from Acros Organics and used as received. B_2pin_2 was kindly provided by AllyChem Co. Ltd. (Dalian, China). Bis-1,3-trifluoromethylbenzene was purchased from ABCR, distilled and degassed before use. $[Ir(COD)(OMe)]_2$ was prepared according to a literature procedure,[1] whereas 2-iodo-1,3-bis(trifluoromethyl)benzene (**I^FXyl**), bis(2,6-bis(trifluoromethyl)phenyl)fluoroborane (**$FB(^FXyl)_2$**) and 9-(4-(bis(2,6-bis(trifluoromethyl)phenyl)boryl)phenyl)-9H-carbazole (**2**) were synthesized using our recently published methodology.[2, 3] All other starting materials were purchased from commercial sources and were used without further purification.

Column chromatography was performed with silica gel 60 (40-63 µ) (purchased from VWR), or alumina 90 active basic (purchased from Merck), and automated flash chromatography was performed on silica gel (Biotage SNAP cartridge KP-Sil 10 g or KP-Sil 25 g), obtained from Biotage, using a Biotage® Isolera Four Flash system. Solvents were generally removed using a rotary evaporator *in vacuo* at a maximum temperature of 55 °C.

NMR spectra were recorded on a Bruker Avance Nanobay HDIII (operating at ^1H: 400 MHz, $^{11}B\{^1H\}$: 128 MHz, $^{19}F\{^1H\}$: 377 MHz) or a Bruker Avance 500 FT NMR spectrometer (operating at ^1H: 500 MHz, $^{11}B\{^1H\}$: 160 MHz, $^{13}C\{^1H\}$: 126 MHz, $^{19}F\{^1H\}$: 470.6 MHz). Chemical shifts (δ) are given in ppm and are referenced to external $BF_3{\cdot}Et_2O$ ($^{11}B\{^1H\}$), $CFCl_3$ ($^{19}F\{^1H\}$). ^1H NMR spectra were referenced via residual proton resonances of $CDCl_3$ (^1H, 7.26 ppm), CD_2Cl_2 (^1H, 5.32 ppm), and C_6D_6 (^1H, 7.16 ppm).[4] $^{13}C\{^1H\}$ spectra are referenced to $CDCl_3$ ($^{13}C\{^1H\}$, 77.16 ppm), CD_2Cl_2 ($^{13}C\{^1H\}$, 53.84 ppm), and C_6D_6 ($^{13}C\{^1H\}$, 128.06 ppm).[4]

GCMS analyses were performed on an Agilent Technologies GCMS system (GC 7890A, EI-MS 5975C).

HRMS were recorded using a Thermo Scientific Exactive Plus Orbitrap MS system with an Atmospheric Sample Analysis Probe (ASAP).

Single-crystal X-ray diffraction: Crystals suitable for single-crystal X-ray diffraction were selected, coated in perfluoropolyether oil, and mounted on MiTeGen sample holders. Diffraction data were collected on Bruker X8 Apex II 4-circle diffractometers with CCD area detectors using Mo-K_α radiation monochromated by graphite (**1**) or multi-layer focusing mirrors (**1-(BPin)$_2$**). Diffraction data of **2-(Bpin)$_2$** were collected on a RIGAKU OXFORD DIFFRACTION XtaLAB Synergy diffractometer with a semiconductor HPA-detector (HyPix-6000) and multi-layer mirror monochromated Cu-K_α radiation. The crystals were

cooled using an open flow N_2 Oxford Cryostream or a Bruker Kryoflex II low-temperature device. Data were collected at 100 K. The images were processed and corrected for Lorentz-polarization effects and absorption as implemented in the Bruker or Rigaku OD (CrysAlis^{Pro}) software packages. The structures were solved using the intrinsic phasing method (SHELXT)[5] and Fourier expansion technique. All non-hydrogen atoms were refined in anisotropic approximation, with hydrogen atoms 'riding' in idealized positions, by full-matrix least squares against F^2 of all data, using SHELXL[6] software and the SHELXLE graphical user interface.[7] The single crystal of **2-(Bpin)₂** was a very thin long needle with long reflections spanning several frames. This led to partly too high reflection intensities from data reduction, which is a result of overlap with other reflections' tails. Though residual values are high, the structure solution can be considered as a proof of the conformation of the molecule. Diamond[8] software was used for graphical representation. Other structural information was extracted using OLEX2[9] software. Crystal data and experimental details are listed in Table S1; full structural information has been deposited with the Cambridge Crystallographic Data Centre: CCDC-1999432 (**1**), 1999433 (**1-(Bpin)$_2$**), and 2003436 (**2-(Bpin)$_2$**).

Photophysical measurements: All measurements were performed in standard quartz cuvettes (1 cm x 1 cm cross-section). UV–visible absorption spectra were recorded using an Agilent 8453 diode array UV-visible spectrophotometer.

Extinction coefficients of **1** and **1-(Bpin)$_2$** were calculated from 6 independently prepared samples in hexane.

Emission spectra were recorded using an Edinburgh Instruments FLSP920 spectrometer equipped with a double monochromator for both excitation and emission, operating in right-angle geometry mode, and all spectra were fully corrected for the spectral response of the instrument. All solutions used for photophysical measurements had a concentration lower than 1×10^{-5} M to minimize inner filter effects during fluorescence measurements.

Fluorescence quantum yields were measured using a calibrated integrating sphere (inner diameter: 150 mm) from Edinburgh Instruments combined with the FLSP920 spectrometer described above. For solution-state measurements, the longest-wavelength absorption maximum of the compound in the respective solvent was chosen as the excitation wavelength.

Fluorescence lifetimes were recorded using the time-correlated single-photon counting (TCSPC) method using the same FLSP920 spectrometer described above. Solutions were excited with a picosecond pulsed diode laser at an emission maximum of 376.6 nm. The full widths at half maximum (FWHM) of the laser pulses were ca. 50–100 ps, while the instrument response function (IRF) had a FWHM of ca. 1.0 ns, measured from the scatter of a Ludox solution at the excitation wavelength. Decays were recorded to at least 10000 counts in the peak channel with a record length of at least 1000 channels. The band pass of the monochromator was adjusted to give a signal count rate of <10 kHz. Iterative reconvolution of the IRF with one decay function and non-linear least-squares analysis were used to analyze the data. The quality of the fit was judged by the calculated value of the reduced χ^2 and visual inspection of the weighted residuals.

Electrochemical measurements: All cyclic voltammetry experiments were conducted in an argon-filled glovebox using a Gamry Instruments Reference 600 potentiostat. A standard

three-electrode cell configuration was employed using a platinum disk working electrode, a platinum wire counter electrode, and a silver wire reference electrode separated by a Vycor frit, serving as the reference electrode. The redox potentials are referenced to the ferrocene/ferrocenium ($[Fc/Fc^+]$) redox couple by using decamethylferrocene ($[Cp^*_2Fe]$; $E_{1/2}$ = –0.532 V in CH_2Cl_2) as an internal standard. Tetra-*n*-butylammonium hexafluorophosphate ($[nBu_4N][PF_6]$) was employed as the supporting electrolyte. Compensation for resistive losses (*iR* drop) was employed for all measurements

Theoretical Studies: All calculations (DFT and TD-DFT) were carried out with the Gaussian 09 (9.E.01)[10] program package and were performed on a parallel cluster system. GaussView (6.0.16), Avogadro[11] and multiwfn[12] were used to visualize the results, to measure calculated structural parameters, and to plot orbital surfaces (isovalue: ± 0.030 $[e\ a_0^{-3}]^{1/2}$). The ground-state geometries were optimized using the B3LYP functional[13] in combination with the 6-31+g(d) basis set.[14, 15] The D3 dispersion correction of Grimme and coworkers was used.[16] The ultrafine integration grid and symmetry constraints were used for all molecules. Frequency calculations were performed on the optimized structure to confirm that it was a local minimum showing no negative (imaginary) frequencies. Based on the optimized structure, the lowest-energy vertical transitions (gas-phase and solvent correction using the polarizable continuum model) were calculated (singlets, 25 states) by TD-DFT, using the Coulomb attenuated functional CAM-B3LYP.[17] The CAM-B3LYP has been shown to describe ICT systems more accurately than B3LYP.[18]

Synthesis

4-(Bis(2,6-bis(trifluoromethyl)phenyl)boryl)-*N*,*N*-diphenylaniline (1)

To a solution of 4-bromo-*N*,*N*-diphenylaniline (510 mg, 1.57 mmol) in hexane (20 mL) *n*BuLi (0.70 mL, 2.3 M, 1.61 mmol) was added dropwise at –78 °C. The reaction was slowly warmed to ambient temperature and stirred overnight. The solution was further heated to 60 °C for 4 h. The suspension was cooled to –78 °C and a solution of bis(2,6-bis(trifluoromethyl)phenyl)fluoroborane (600 mg, 1.31 mmol) in MTBE (5 mL) was added dropwise. The reaction was warmed to room temperature and stirred for 12 h. All volatiles were removed *in vacuo*. The residue was dissolved in hexane and filtered over a short silica plug. The product was eluted with Et_2O and purified by column chromatography (silica, hexane/CH_2Cl_2 (9:1)). Recrystallization from hexane gave 4-(bis(2,6-bis(trifluoromethyl)phenyl)boryl)-*N*,*N*-diphenylaniline (**1**) as bright greenish-yellow crystals in 95 % yield (852 mg, 1.25 mmol).

^{1}H NMR (400 MHz, $CDCl_3$): δ(ppm) = 7.93 (d, ^3J = 8 Hz, 4H), 7.69 (t, ^3J = 8 Hz, 2H), 7.35 – 7.28 (m, 4H), 7.20 – 7.05 (m, 6H), 7.01 (d, ^3J = 9 Hz, 2H), 6.87 (d, ^3J = 9 Hz, 2H).

^{13}C{^{1}H} NMR (126 MHz, $CDCl_3$): δ(ppm) = 152.1 (C_q, 1C), 146.7 (C_q, 2C), 139.9 (C_q, 4C), 139.6 (C_q, 2C), 138.7 (CH, 2C), 129.9 (CH, 2C), 129.6 (CH, 4C), 126.1 (CH, 4C), 124.6 (CH, 2C), 124.1 (C_q, q, J_{CF} = 273 Hz, 4C), 118.7 (CH, 2C).

^{11}B NMR (128 MHz, CD_2Cl_2): δ(ppm) = 72.0 (s, br).

^{19}F NMR (377 MHz, CD_2Cl_2): δ(ppm) = –56.12 (s, br), 51.06 (s, br).

Elem. Anal.: Calc. (%) for $C_{34}H_{20}BF_{12}N$: C 59.94, H 2.96, N 2.06; found: C 59.95, H 3.02, N 2.29.

HRMS (APCI pos): calc.: 682.1570 [m/z]; found: 682.1564 [MH^+]

4-(Bis(4-(4,4,5,5-tetramethyl-1,3,2-dioxaborolan-2-yl)-2,6-bis(trifluoromethyl)phenyl)boryl)-N,N-diphenylaniline (1-(Bpin)$_2$)

A solution of B_2pin_2 (56 mg, 220 µmol), [Ir(COD)(OMe)]$_2$ (1.5 mg, 2.20 µmol), and dtbpy (1.26 mg, 4.40 µmol) in hexane (40 mL) was stirred for 10 min at room temperature. Then, 4-(bis(2,6-bis(trifluoromethyl)phenyl)boryl)-*N*,*N*-diphenylaniline (100 mg, 147 µmol) was added and the reaction was stirred at room temperature for 72 h. All volatiles were removed *in vacuo* and the residue was washed with MeOH (3 x 30 mL). The product was extracted with Et_2O and all volatiles were removed *in vacuo* to give 4-(bis(4-(4,4,5,5-tetramethyl-1,3,2-dioxaborolan-2-yl)-2,6-bis(trifluoromethyl)phenyl)boryl)-N,N-diphenylaniline (**1-(Bpin)$_2$**) as bright greenish-yellow crystals in 90 % yield (123 mg, 132 µmol).

^{1}H NMR (500 MHz, C_6D_6): δ(ppm) = 8.72 (s, 4H), 6.97 – 7.04 (m, 10H), 6.82 – 6.85 (m, 4H), 1.07 (s, 24H).

^{1}H NMR (400 MHz, CD_2Cl_2): δ(ppm) = 8.28 (s, 4H), 7.34- 7.28 (m. 4 H), 7.17 – 7.09 (m, 6H), 6.97 – 6.93 (m, 2H), 6.84 – 6.80 (m, 2H), 1.37 (s, 24 H).

^{13}C{^{1}H} NMR (126 MHz, C_6D_6): δ(ppm) = 152.6 (C_q, 1C), 146.9 (C_q, 2C), 143.2 (C_q, 2C), 139.8 (CH, 1C), 139.4 (CH, 2C), 136.0 (CH, br, 4C, assigned by HSQC), 131.9 (C_q, 4C), 129.8 (CH, 4C), 126.6 (CH, 4C), 125.0 (C_q, q, $^1J_{CF}$ = 273 Hz, 4 C) 124.8 (CH, 2C), 118.8 (CH, 2C), 84.8 (C_q, 4C), 24.5 (CH_3, 8C).

^{11}B NMR (128 MHz, CD_2Cl_2): δ(ppm) = 68.9 (s, br), 30.6 (s, br).

^{19}F NMR (377 MHz, CD_2Cl_2): δ(ppm) = –50.96 (s, br). –56.12(s, br).

Elem. Anal.: Calc. (%) for $C_{46}H_{42}B_3F_{12}NO_4$: C 59.20, H 4.54, N 1.50; found: C 59.54, H 4.68, N 1.55.

9-(4-(Bis(4-(4,4,5,5-tetramethyl-1,3,2-dioxaborolan-2-yl)-2,6-bis(trifluoromethyl)phenyl)boryl)phenyl)-9H-carbazole (2-(Bpin)$_2$)

A solution of B_2pin_2 (656 mg, 2.58 mmol), $[Ir(COD)(OMe)]_2$ (30.2 mg, 45.6 µmol), and dtbpy (26.1 mg, 91.2 µmol, 0.06 eq.) in hexane (40 mL) was stirred for 10 min at room temperature. Then 9-(4-(bis(2,6-bis(trifluoromethyl)phenyl)boryl)phenyl)-9H-carbazole (**2**) (1.03 g, 1.52 mmol) was added and the reaction was stirred at room temperature for 1 d. After removing all volatiles *in vacuo* the residue was washed on a silica plug with hexane (500 mL), then the plug was extracted with hexane/Et_2O (9:1). After removing all volatiles *in vacuo*, the product was recrystallized from hexane to give 9-(4-(bis(4-(4,4,5,5-tetramethyl-1,3,2-dioxaborolan-2-yl)-2,6-bis(trifluoromethyl)phenyl)boryl)phenyl)-9H-carbazole (**2-(Bpin)$_2$**) as colorless blue-greenish fluorescent crystals in 78 % yield (1.11 g, 1.19 mmol).

^{1}H NMR (500 MHz, C_6D_6): δ(ppm) = 8.74 (s, 4H), 8.03 – 8.00 (m, 2H), 7.49 – 7.45 (m, 2H), 7.30 – 7.26 (m, 4H), 7.24 – 7.18 (m, 4H), 1.07 (s, 24H).

^{13}C{^{1}H} NMR (126 MHz, C_6D_6): δ(ppm) = 145.0 (C_q, 1C), 142.2 (C_q, 1C), 142.0 (C_q, 2C), 140.8 (C_q, 2C), 138.6 (CH, 2C), 136.4 (CH, 4C), 132.8 (C_q, 2C), 126.6 (CH, 2C), 125.5 (CH, 2C), 124.8 (C_q, q, $^1J_{CF}$ = 247 Hz), 124.4 (C_q, 2C), 120.9 (CH, 2C), 120.7 (CH, 2C), 110.3 (CH, 2C), 84.9 (C_q, 4C), 24.8 (CH_3, 8C).

^{11}B NMR (160 MHz, C_6D_6): δ(ppm) = 31.9 (s, br, Bpin).

^{19}F NMR (470 MHz, C_6D_6): δ(ppm) = –52.8 (s, br).

Elem. Anal.: Calc. (%) for $C_{46}H_{40}B_3F_{12}NO_4$: C 59.33, H 4.33, N 1.50; found: C 59.67, H 4.40, N 1.55.

NMR Spectra

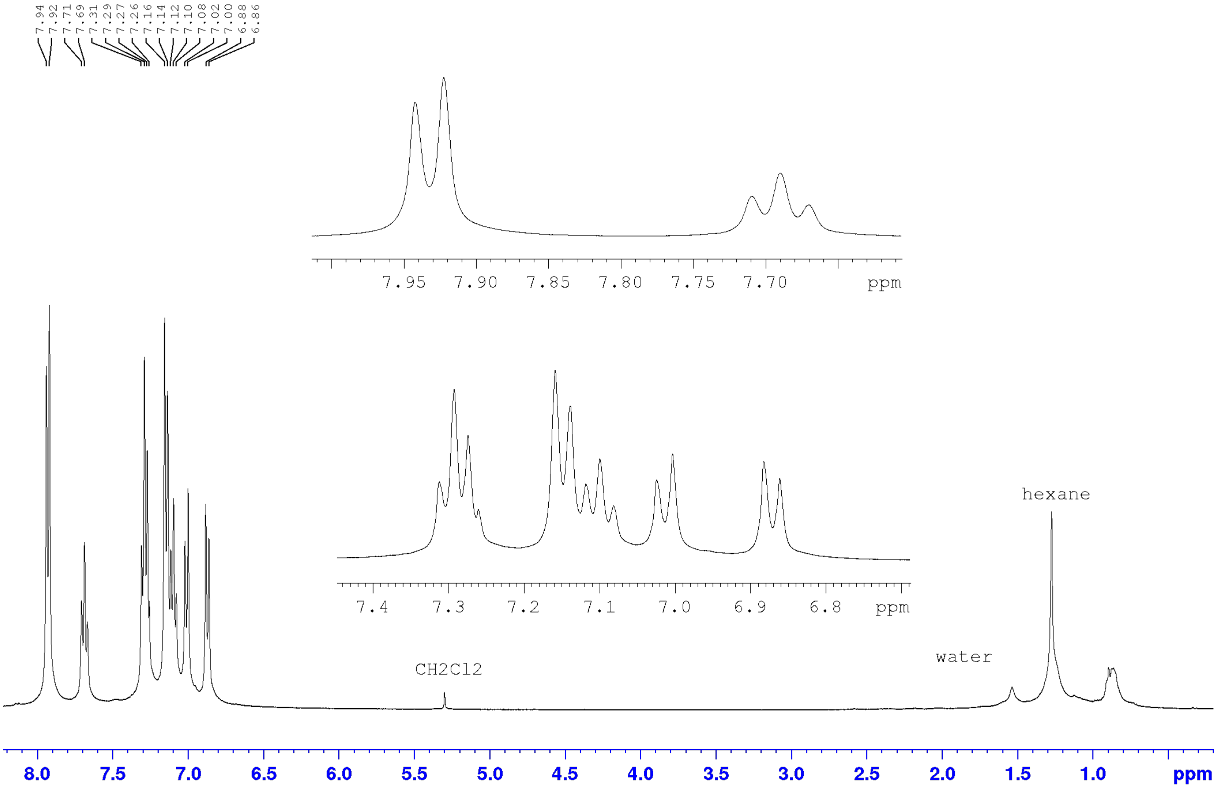

Figure S1: ^{1}H NMR spectrum (400 MHz, 298 K, $CDCl_3$) of 4-(bis(2,6-bis(trifluoromethyl)phenyl)boryl)-*N*,*N*-diphenylaniline (**1**).

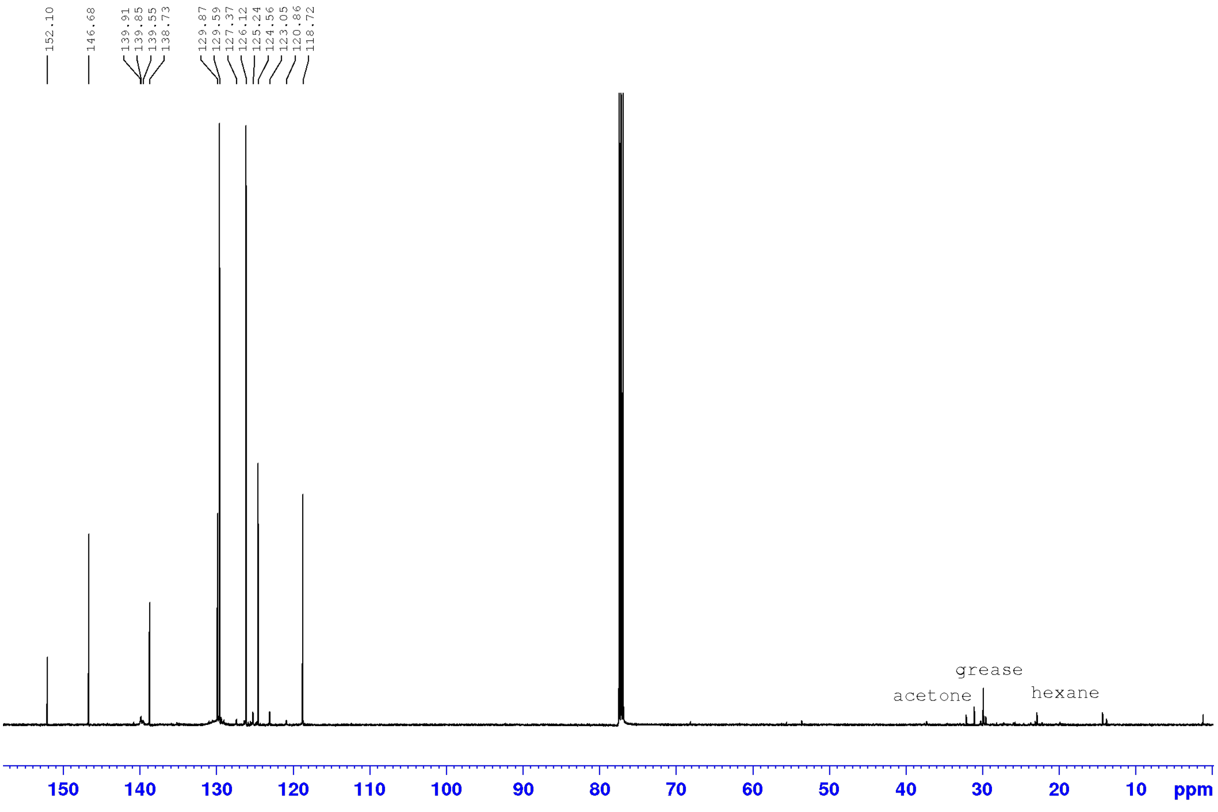

Figure S2: ^{13}C {^{1}H} NMR spectrum (126 MHz, 298 K, $CDCl_3$) of 4-(bis(2,6-bis(trifluoromethyl)phenyl)boryl)-*N*,*N*-diphenylaniline (**1**).

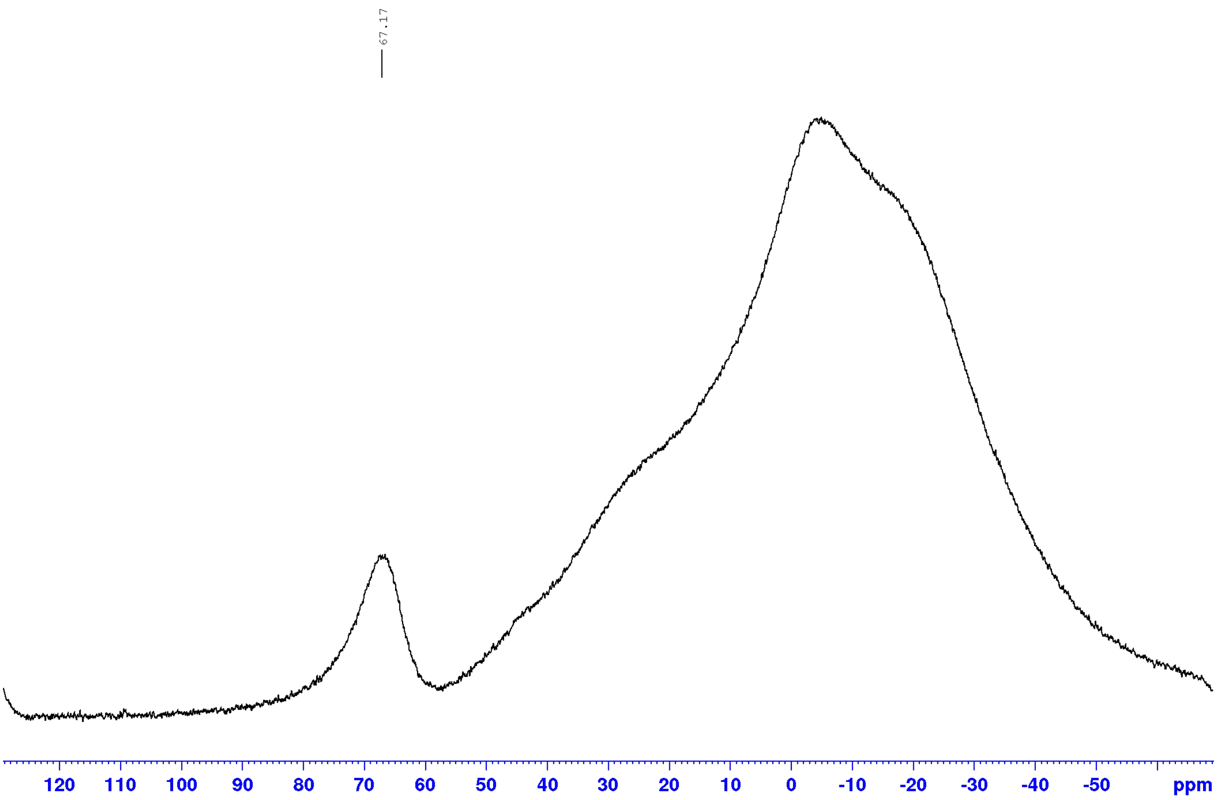

Figure S3: ^{11}B NMR spectrum (128 MHz, 298 K, CD_2Cl_2) of 4-(bis(2,6-bis(trifluoromethyl)phenyl)boryl)-*N*,*N*-diphenylaniline (**1**).

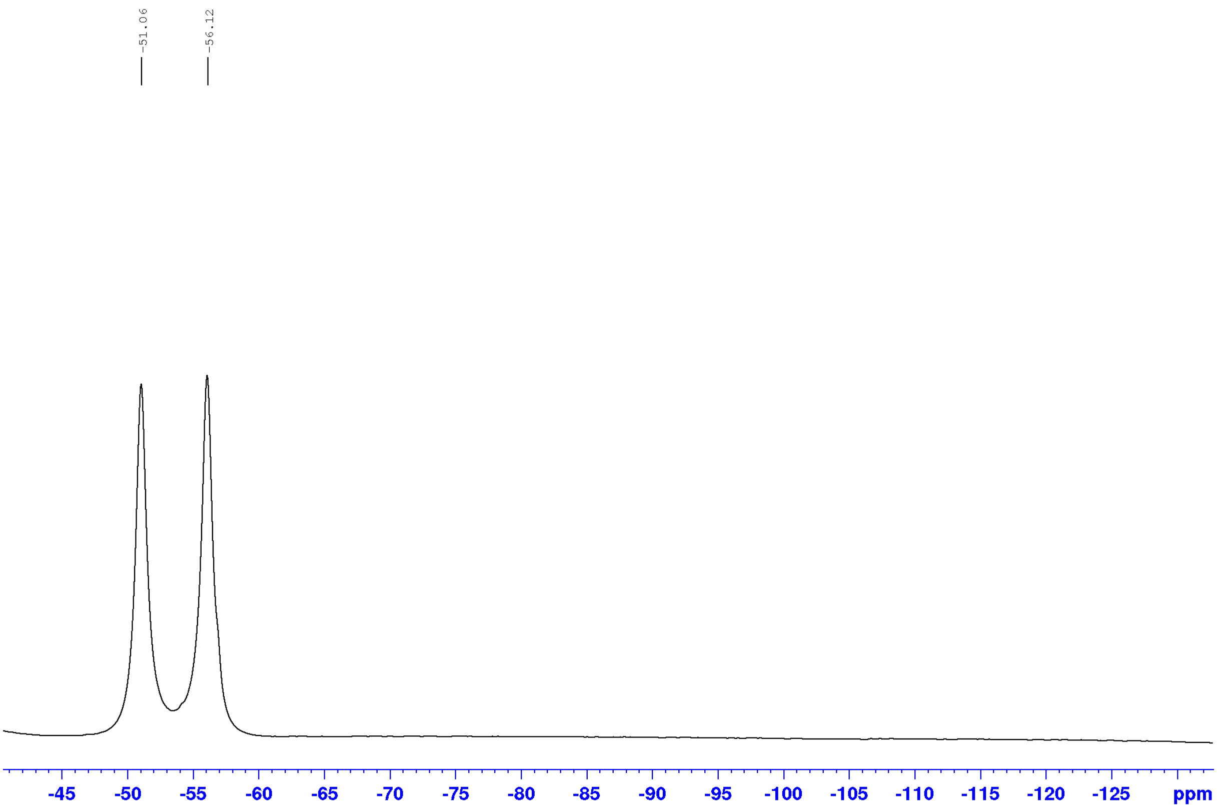

Figure S4: ^{19}F NMR spectrum (377 MHz, 298 K, CD_2Cl_2) of 4-(bis(2,6-bis(trifluoromethyl)phenyl)boryl)-*N*,*N*-diphenylaniline (**1**).

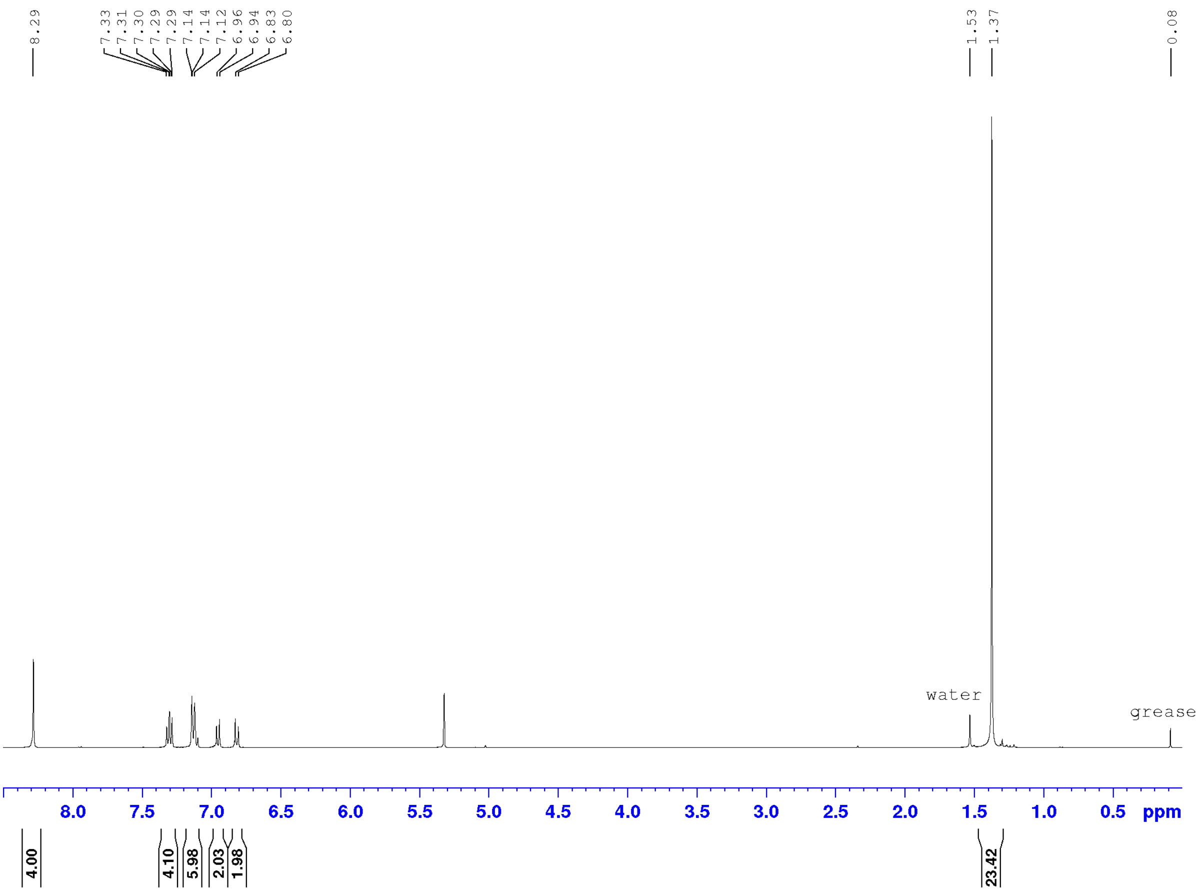

Figure S5: ^{1}H NMR spectrum (400 MHz, 298 K, CD_2Cl_2) of 4-(bis(4-(4,4,5,5-tetramethyl-1,3,2-dioxaborolan-2-yl)-2,6-bis(trifluoromethyl)phenyl)boryl)-N,N-diphenylaniline (**1-(Bpin)$_2$**).

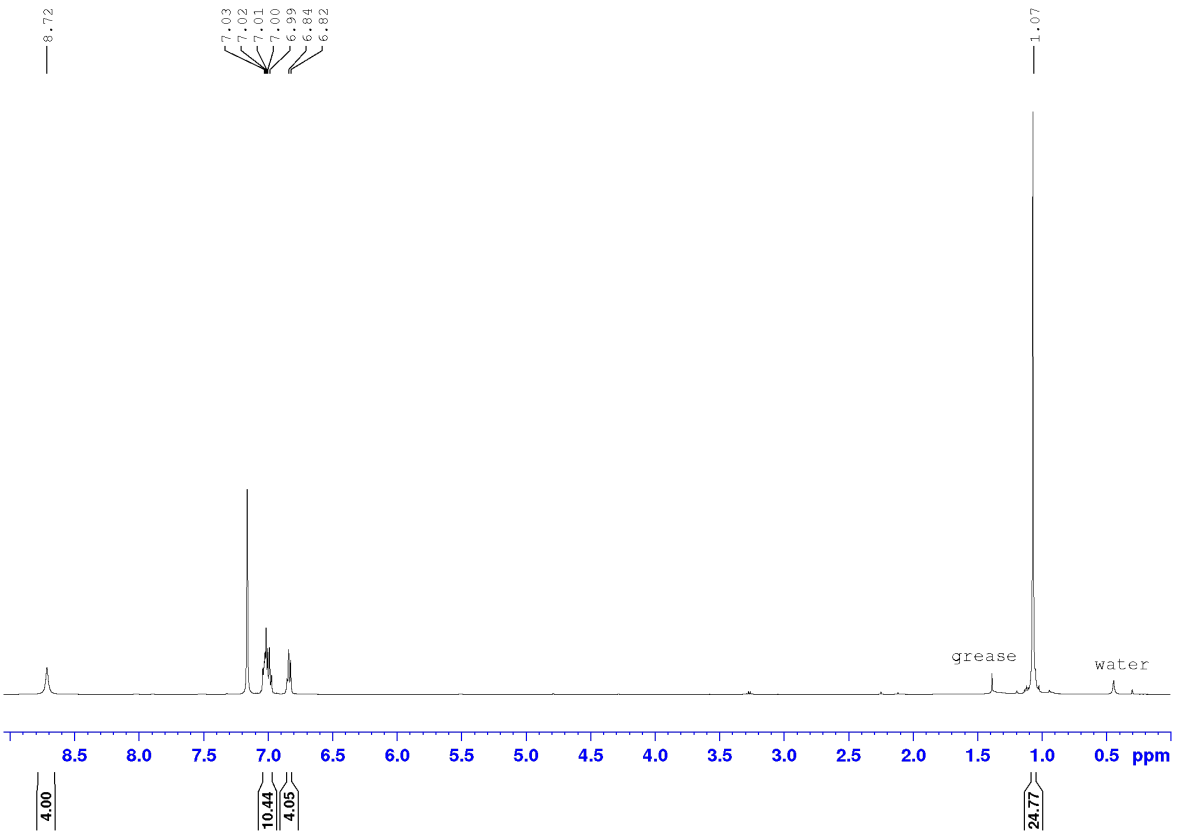

Figure S6: ^{1}H NMR spectrum (500 MHz, 298 K, C_6D_6) of 4-(bis(4-(4,4,5,5-tetramethyl-1,3,2-dioxaborolan-2-yl)-2,6-bis(trifluoromethyl)phenyl)boryl)-N,N-diphenylaniline (**1-(Bpin)$_2$**).

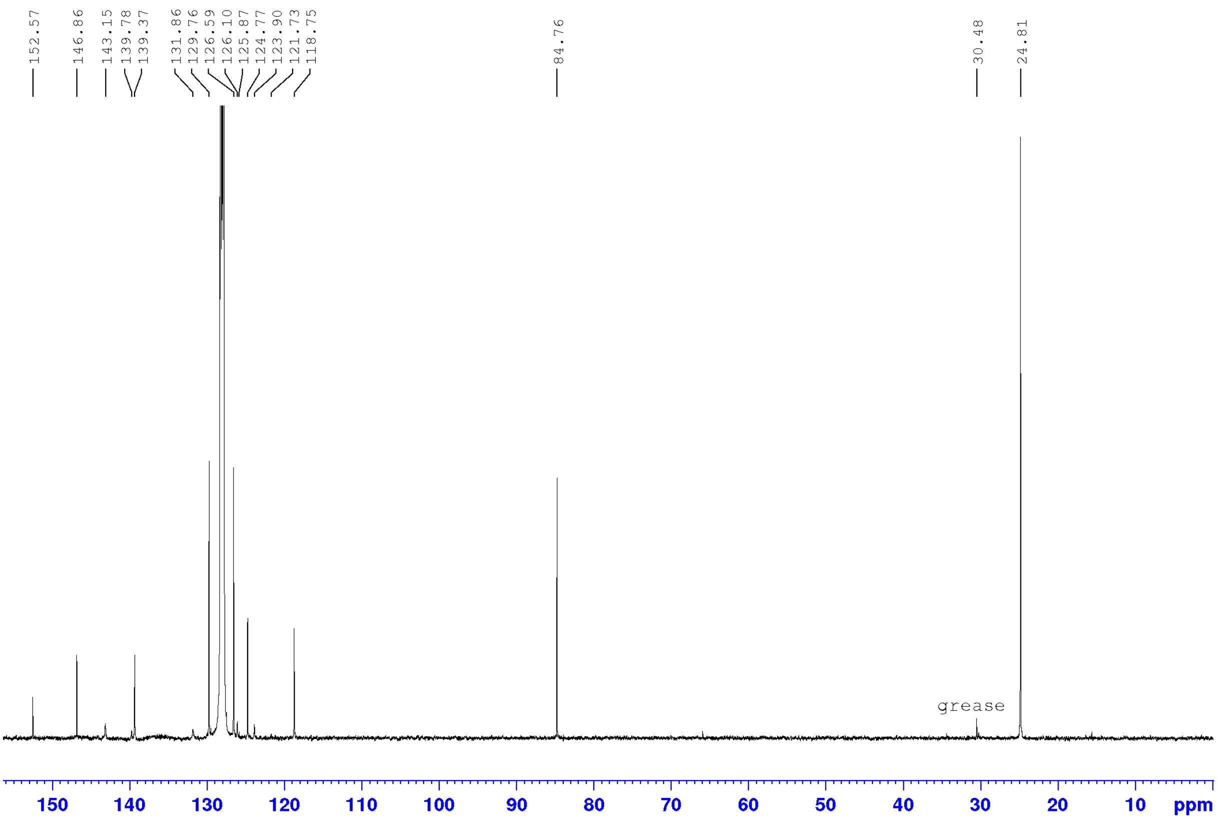

Figure S7: ^{13}C {^{1}H} NMR spectrum (126 MHz, 298 K, $CDCl_3$) of 4-(bis(4-(4,4,5,5-tetramethyl-1,3,2-dioxaborolan-2-yl)-2,6-bis(trifluoromethyl)phenyl)boryl)-N,N-diphenylaniline (**1-(Bpin)₂**).

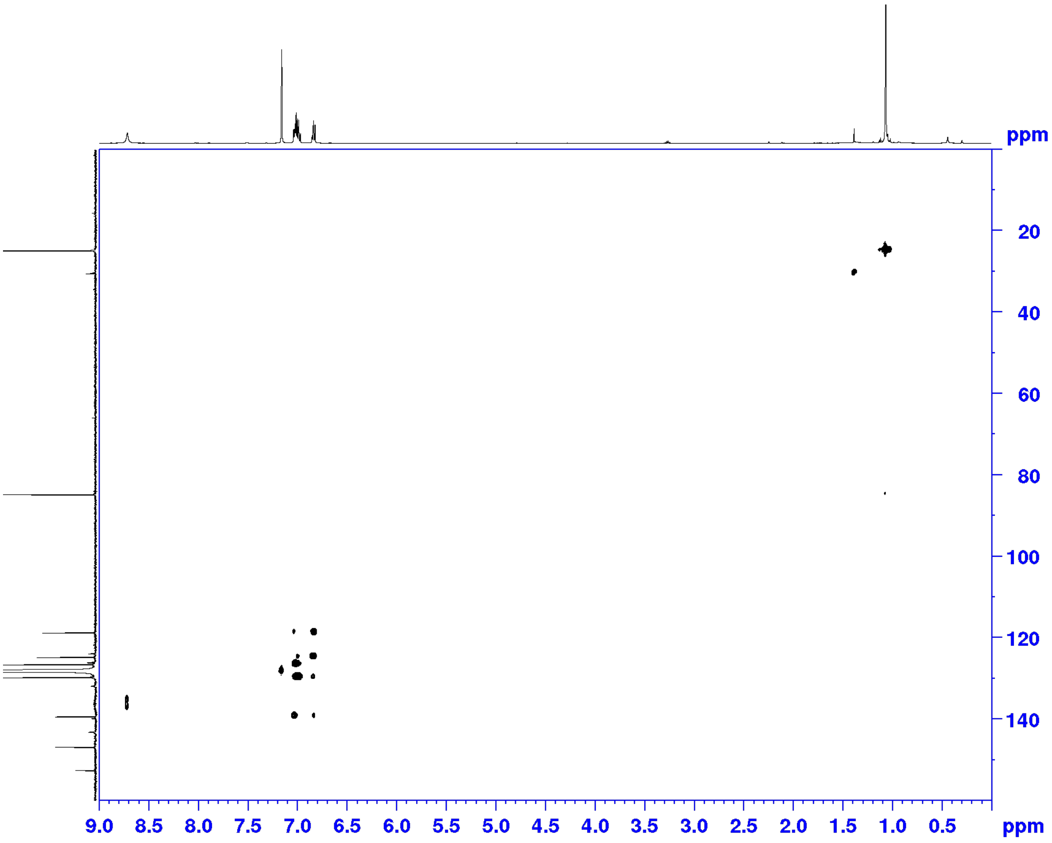

Figure S8: HSQC NMR spectrum (^{13}C{^{1}H} 126 MHz, ^{1}H 500 MHz, 298 K, $CDCl_3$) of 4-(bis(4-(4,4,5,5-tetramethyl-1,3,2-dioxaborolan-2-yl)-2,6-bis(trifluoromethyl)phenyl)boryl)-N,N-diphenylaniline (**1-(Bpin)$_2$**).

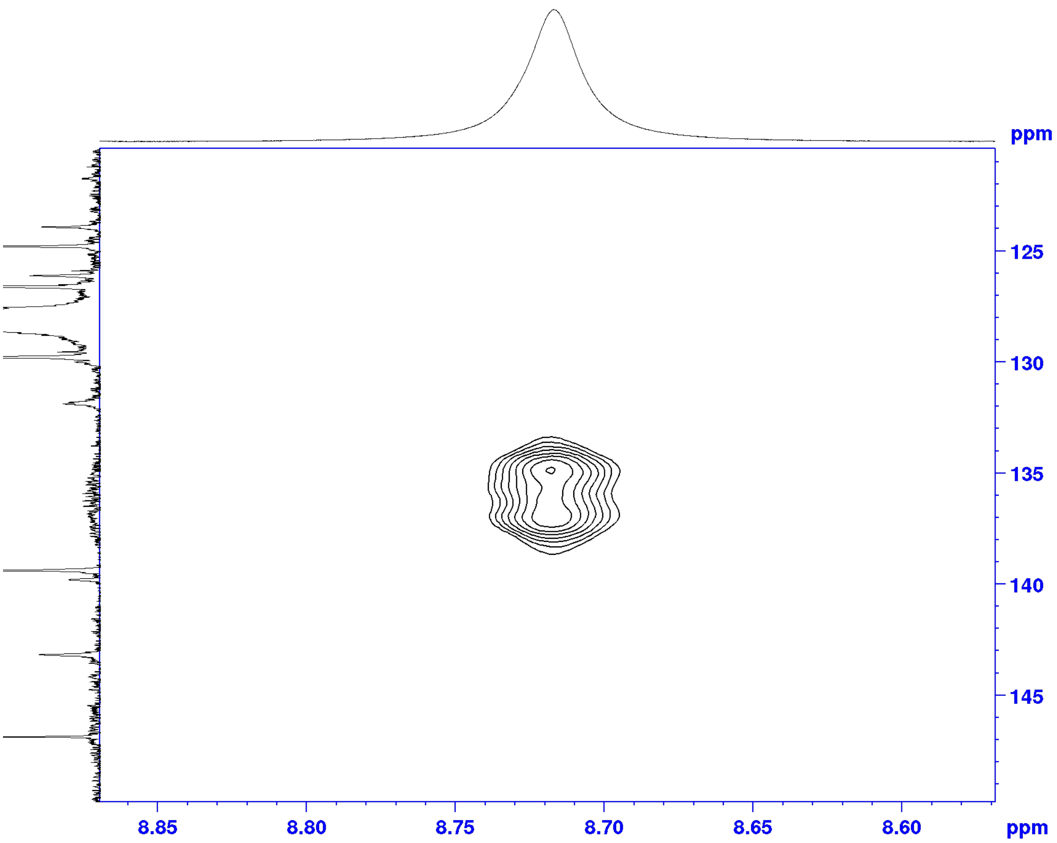

Figure S9: HSQC NMR spectrum ($^{13}C\{^{1}H\}$ 126 MHz, ^{1}H 500 MHz, 298 K, $CDCl_3$) of 4-(bis(4-(4,4,5,5-tetramethyl-1,3,2-dioxaborolan-2-yl)-2,6-bis(trifluoromethyl)phenyl)boryl)-N,N-diphenylaniline (**1-(Bpin)$_2$**).

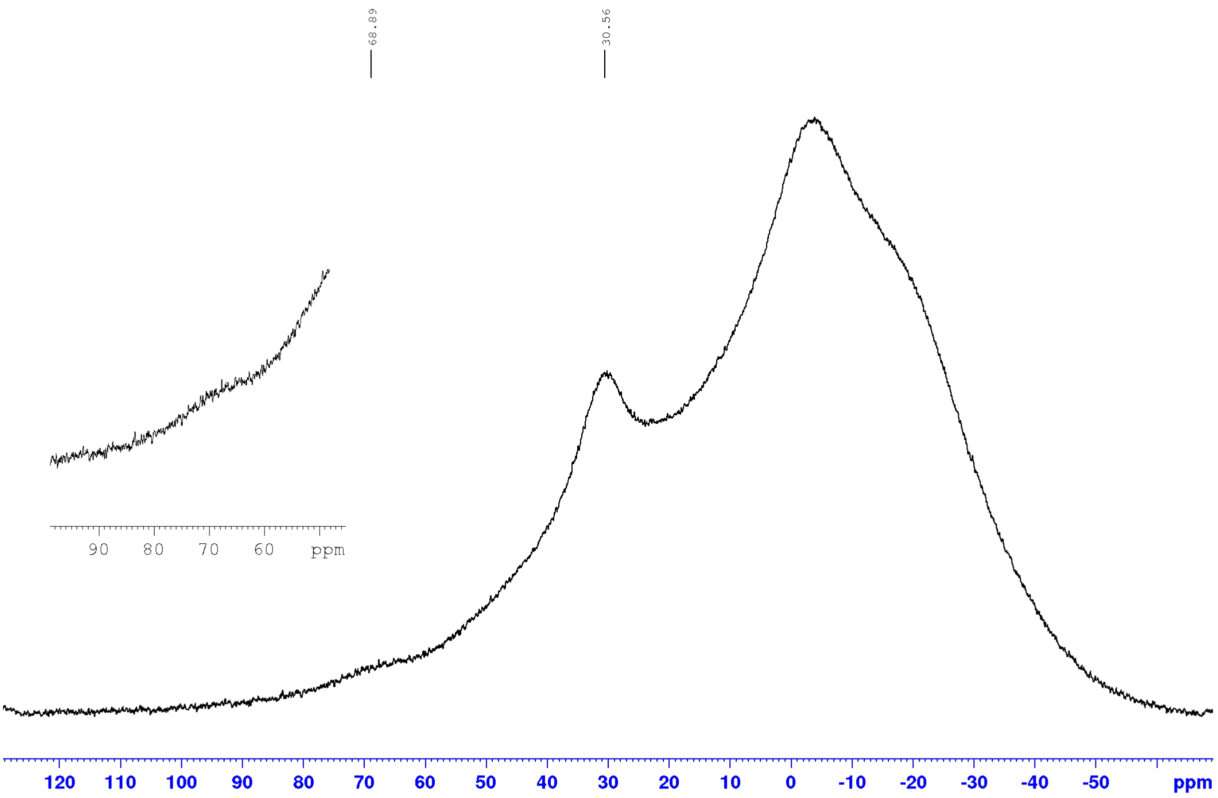

Figure S10: ^{11}B NMR spectrum (128 MHz, 298 K, CD_2Cl_2) of 4-(bis(4-(4,4,5,5-tetramethyl-1,3,2-dioxaborolan-2-yl)-2,6-bis(trifluoromethyl)phenyl)boryl)-N,N-diphenylaniline (**1-(Bpin)$_2$**).

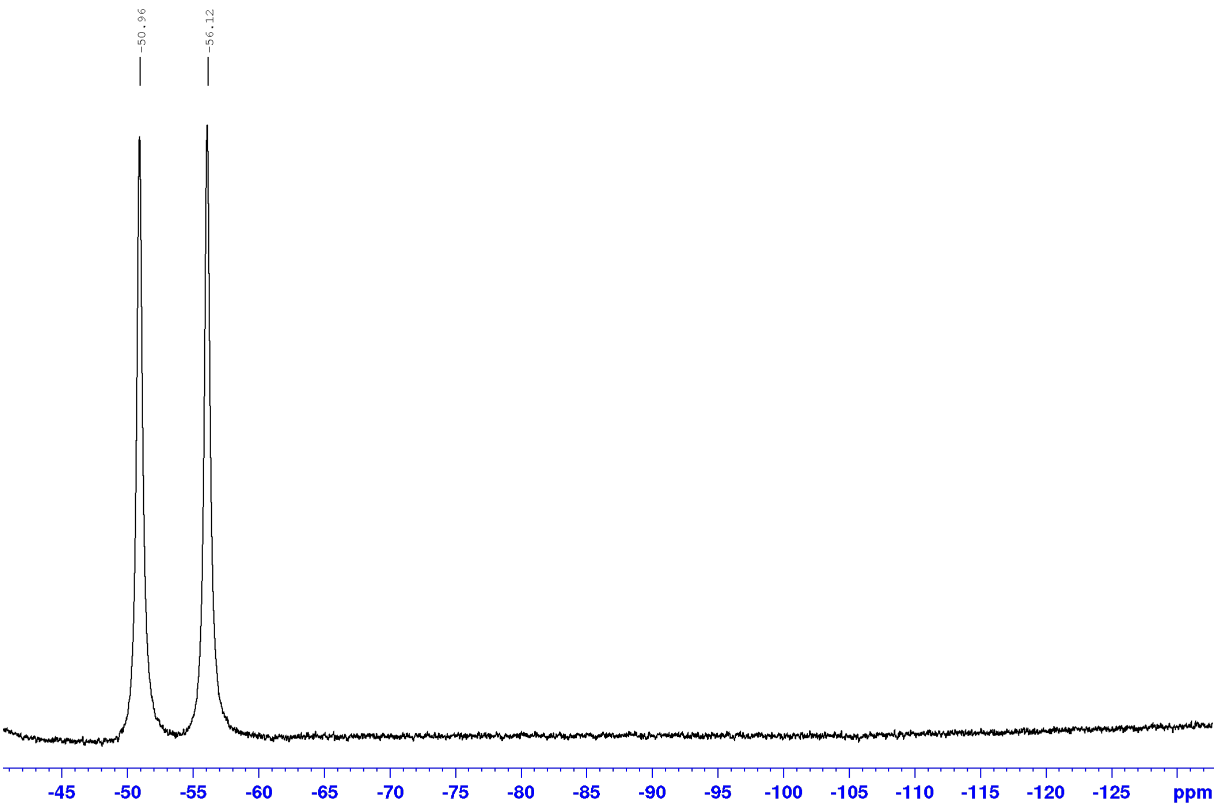

Figure S11: ^{19}F NMR spectrum (377 MHz, 298 K, CD_2Cl_2) of 4-(bis(4-(4,4,5,5-tetramethyl-1,3,2-dioxaborolan-2-yl)-2,6-bis(trifluoromethyl)phenyl)boryl)-N,N-diphenylaniline (**1-(Bpin)₂**).

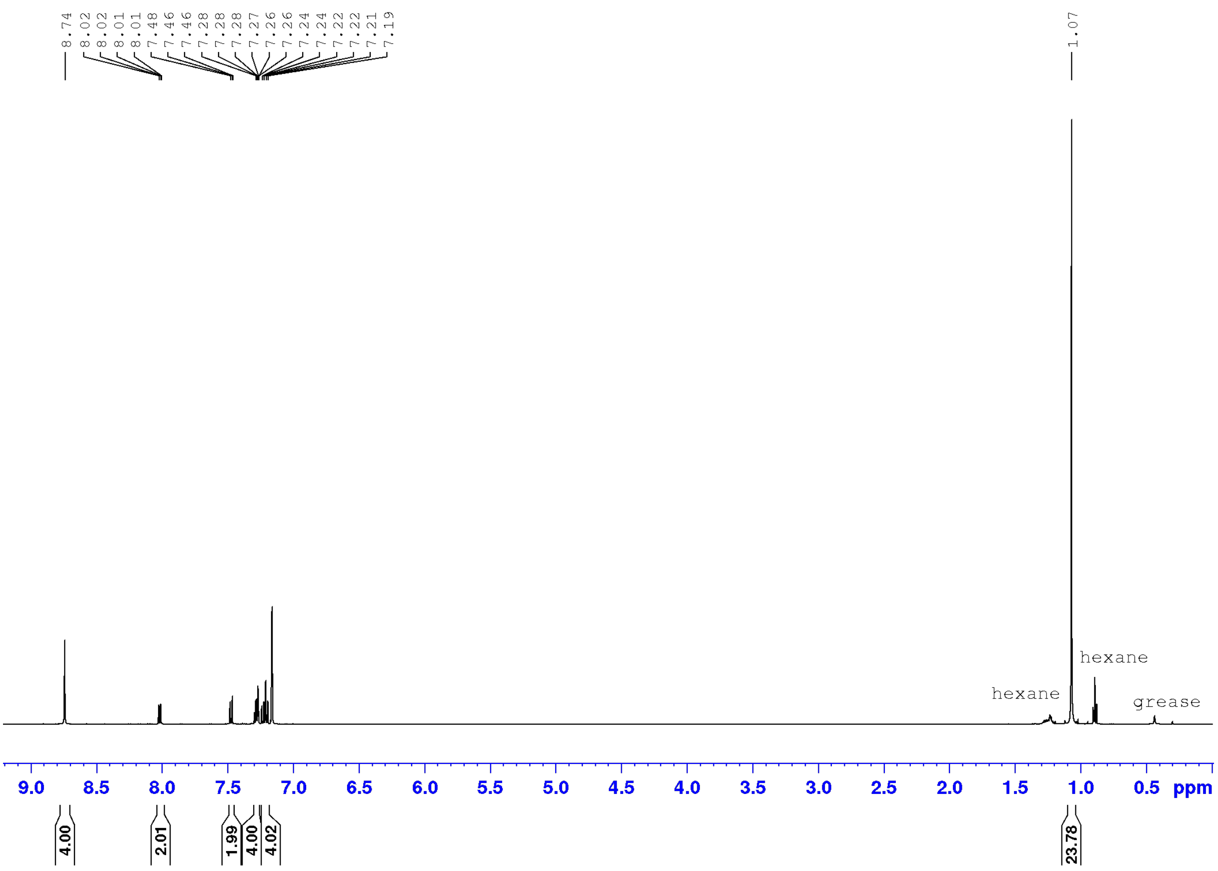

Figure S12: ^{1}H NMR spectrum (500 MHz, 298 K, C_6D_6) of 9-(4-(Bis(4-(4,4,5,5-tetramethyl-1,3,2-dioxaborolan-2-yl)-2,6-bis(trifluoromethyl)phenyl)boryl)phenyl)-9H-carbazole (**2-(Bpin)₂**).

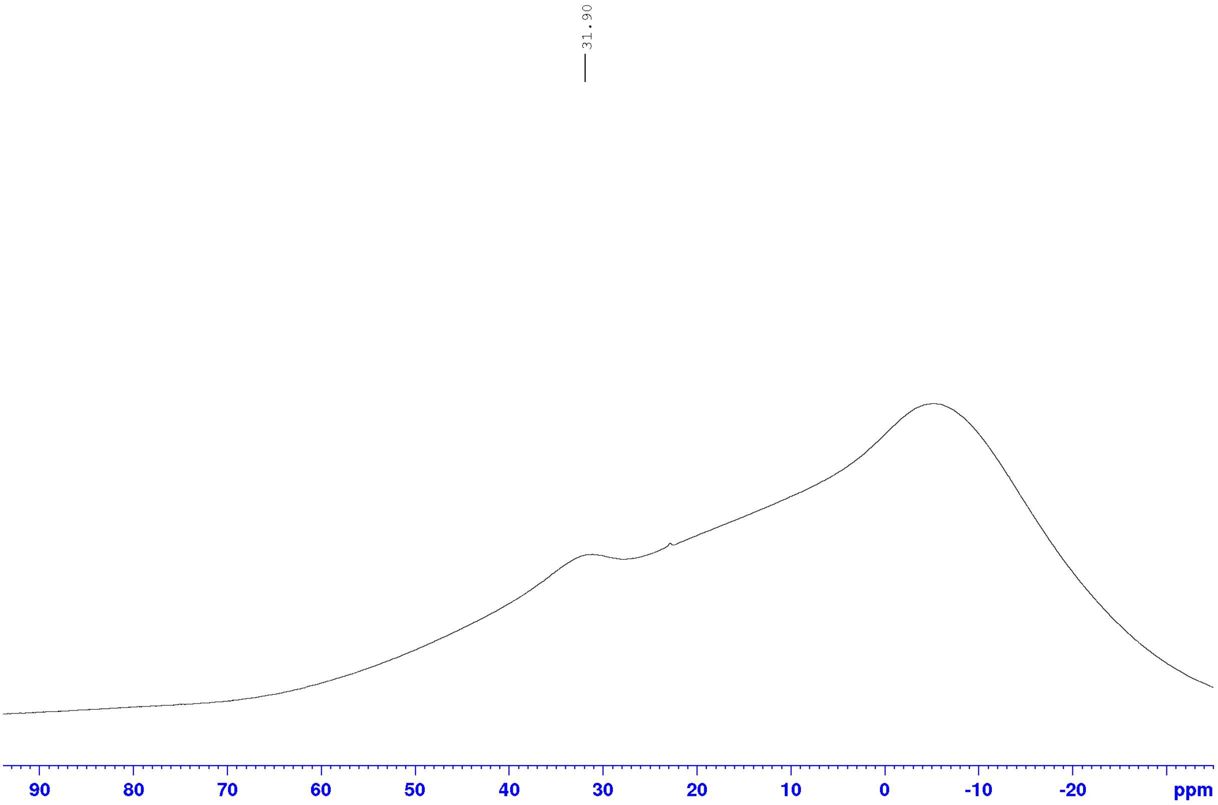

Figure S13: ^{11}B NMR spectrum (160 MHz, 298 K, C_6D_6) of 9-(4-(Bis(4-(4,4,5,5-tetramethyl-1,3,2-dioxaborolan-2-yl)-2,6-bis(trifluoromethyl)phenyl)boryl)phenyl)-9H-carbazole (**2-(Bpin)$_2$**).

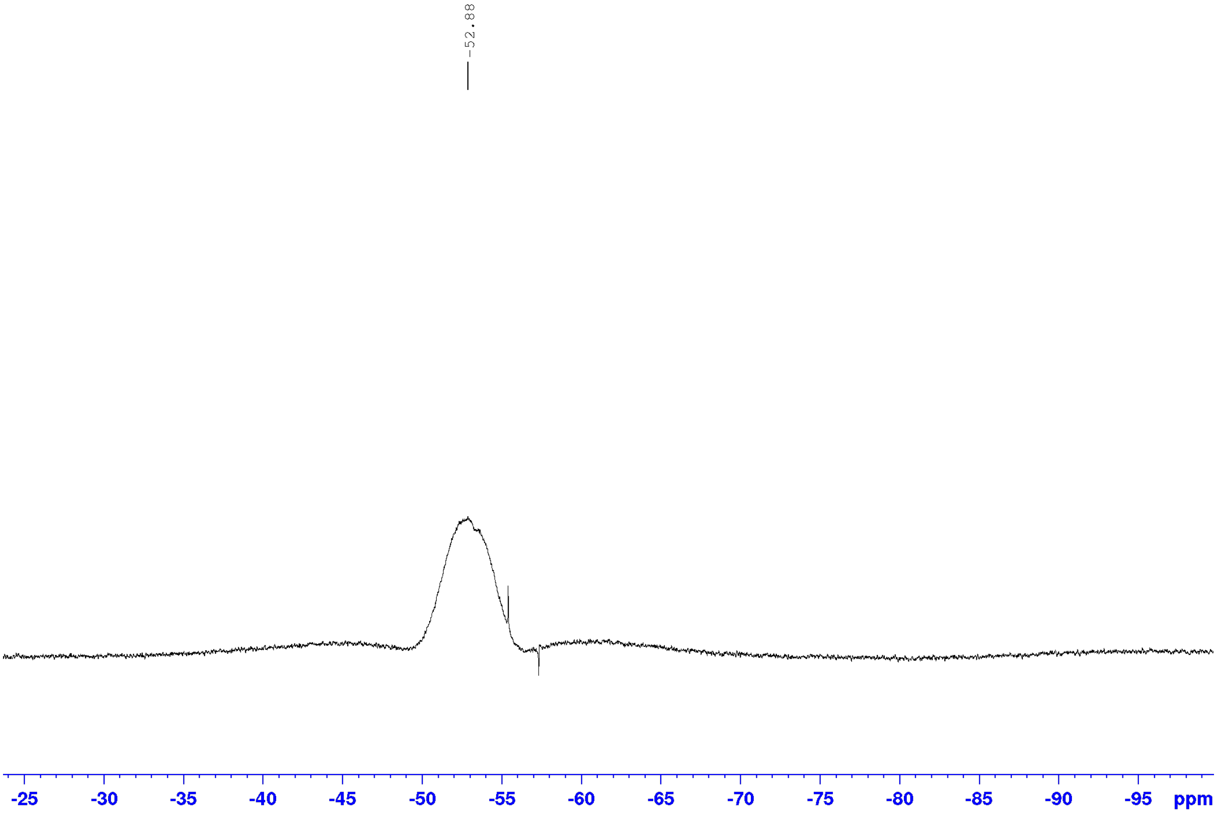

Figure S14: ^{19}F NMR spectrum (470 Hz, 298 K, C_6D_6) of 9-(4-(Bis(4-(4,4,5,5-tetramethyl-1,3,2-dioxaborolan-2-yl)-2,6-bis(trifluoromethyl)phenyl)boryl)phenyl)-9H-carbazole (**2-(Bpin)$_2$**).

Crystal structure determinations

Table S1: Single-crystal X-ray diffraction data and structure refinements of **1** and **1-(Bpin)$_2$**.

Data	**1**	**1-(Bpin)$_2$**
CCDC number	1999432	1999433
Empirical formula	$C_{34}H_{20}BF_{12}N$	$C_{46}H_{42}B_3F_{12}NO_4$
Formula weight (g·mol^{-1})	681.32	933.23
Temperature (K)	100(2)	100(2)
Radiation, λ (Å)	Mo-K$_\alpha$ 0.71073	Mo-K$_\alpha$ 0.71073
Crystal size (mm^3)	0.45×0.40×0.05	0.35×0.25×0.08
Crystal color, habit	Yellow plate	Yellow plate
Crystal system	Monoclinic	Monoclinic
Space group	$P2_1/n$	$C2/c$
a (Å)	8.756(4)	26.351(9)
b (Å)	35.52(3)	14.031(5)
c (Å)	9.607(4)	13.994(5)
α (°)	90	90
β (°)	101.430(12)	121.024(18)
γ (°)	90	90
Volume (Å^3)	2929(3)	4434(3)
Z	4	4
ρ_{cal} (g·cm^{-3})	1.545	1.398
μ (mm^{-1})	0.143	0.121
F(000)	1376	1920
θ range (°)	2.441 – 25.999	1.709 – 26.000
Reflections collected	19617	20936
Unique reflections	5747	4377
Min. / max. transmission	0.6246/0.7454	0.6778/0.7454
Parameters / restraints	433 / 0	363 / 151
GooF on F^2	1.037	1.022
R_1 [I>2σ(I)]	0.0439	0.0486
wR^2 (all data)	0.1033	0.1437
Max. / min. residual electron density (e·Å^{-3})	0.248 / –0.293	0.560 / –0.278

-Table S1 cont.-

Data	**2-(Bpin)$_2$**
CCDC number	2003436
Empirical formula	$C_{46}H_{40}B_3F_{12}NO_4$
Formula weight (g·mol^{-1})	931.22
Temperature (K)	100(2)
Radiation, l (Å)	Cu-K$_a$ 1.54184
Crystal size (mm^3)	0.57×0.06×0.04
Crystal color, habit	Colorless needle
Crystal system	Monoclinic
Space group	$P2_1/n$
a (Å)	19.3861(10)
b (Å)	10.3578(2)
c (Å)	24.2273(10)
a (°)	90
b (°)	107.664(5)
g (°)	90
Volume (Å^3)	4635.4(4)
Z	4
ρ_{cal} (g·cm^{-3})	1.334
μ (mm^{-1})	1.005
F(000)	1912
θ range (°)	2.570 – 67.079
Reflections collected	29926
Unique reflections	8166
Min. / max. transmission	0.511/1.000
Parameters / restraints	669 / 168
GooF on F^2	1.078
R$_1$ [I>2s(I)]	0.0965
wR^2 (all data)	0.2683
Max. / min. residual electron density (e·Å^{-3})	0.419 / –0.361

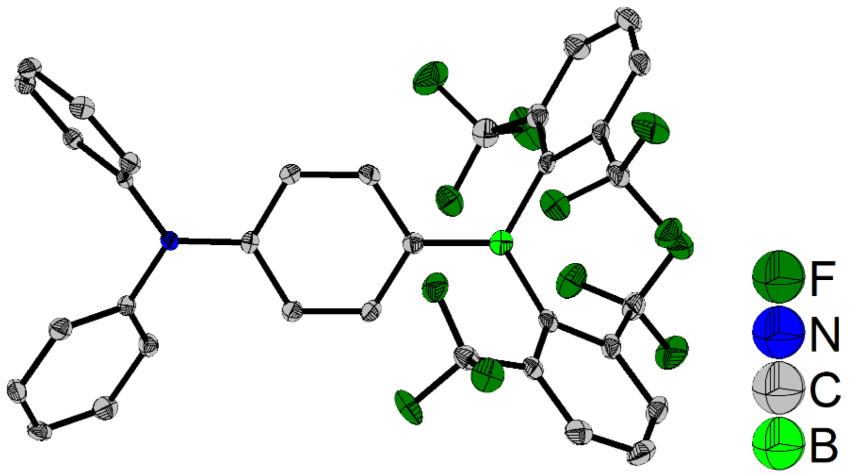

Figure S15: The solid-state molecular structure of **1** determined by single-crystal X-ray diffraction at 100 K. All ellipsoids are drawn at the 50% probability level. H atoms and solvent molecules are omitted for clarity.

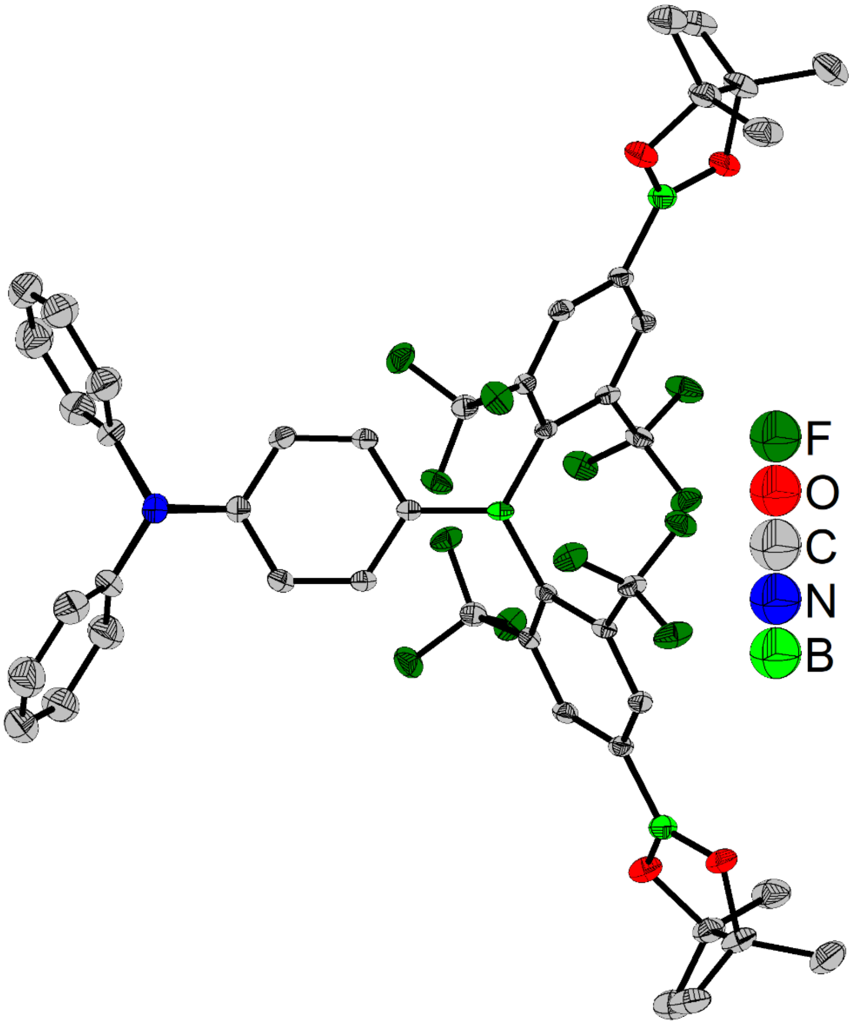

Figure S16: The solid-state molecular structure of **1-(Bpin)₂** determined by single-crystal X-ray diffraction at 100 K. All ellipsoids are drawn at the 50% probability level. H atoms and solvent molecules are omitted for clarity. The diphenylamine group (NPh_2) is disordered by twofold rotational symmetry and only one part (50%) is shown here.

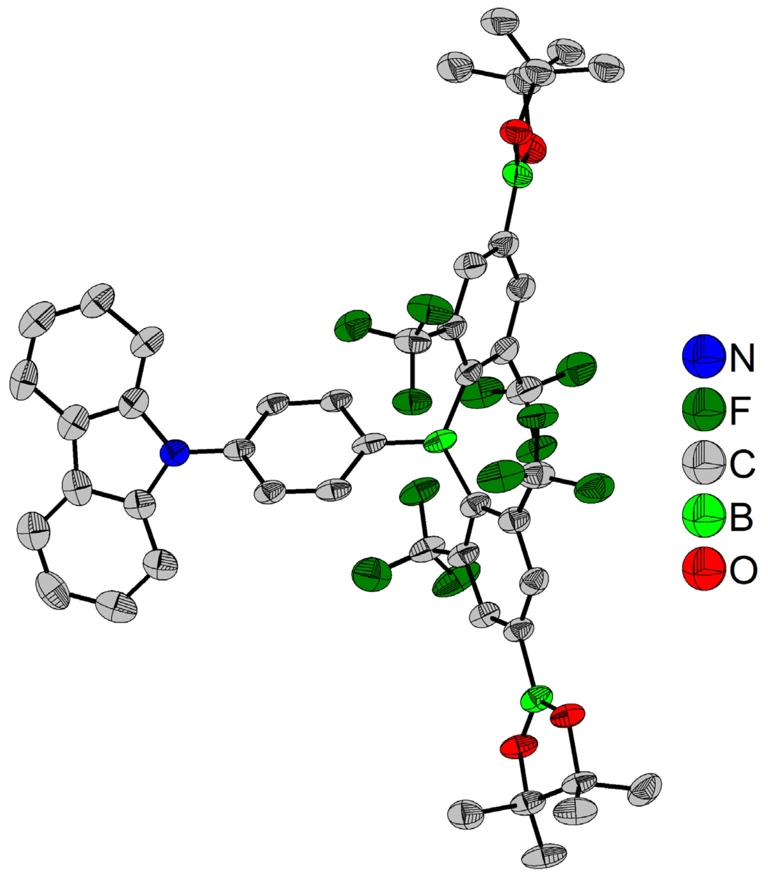

Figure S17: The solid-state molecular structure of **2-(Bpin)$_2$** determined by single-crystal X-ray diffraction at 100 K. All ellipsoids are drawn at the 50% probability level. H atoms are omitted for clarity. One of the Bpin groups is disordered and only the major part (71%) is shown here.

Photophysical data

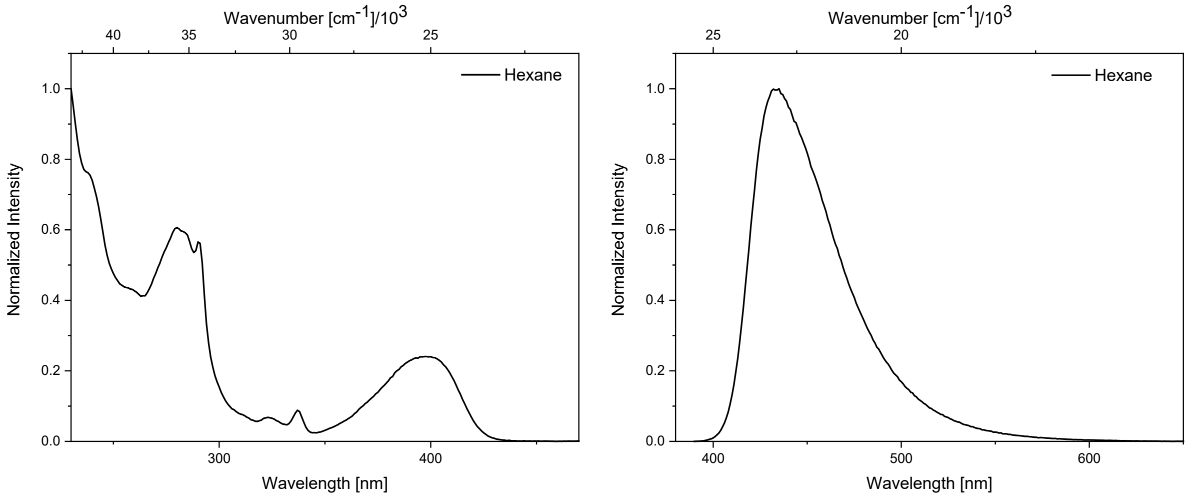

Figure S18: Normalized absorption (left) and emission (right) spectra of **2-Bpin$_2$** in hexane.

Electrochemistry

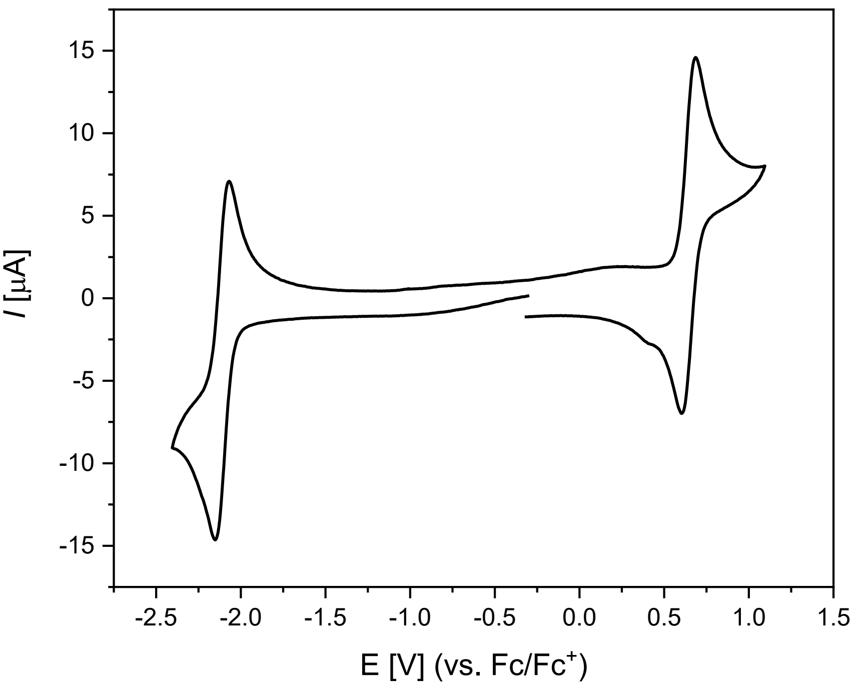

Figure S19: Cyclic voltammogram of **1** in CH_2Cl_2 with [nBu$_4$N][PF$_6$] as the electrolyte and a scan rate of 250 mVs^{-1} referenced vs. the Fc/Fc$^+$ redox couple.

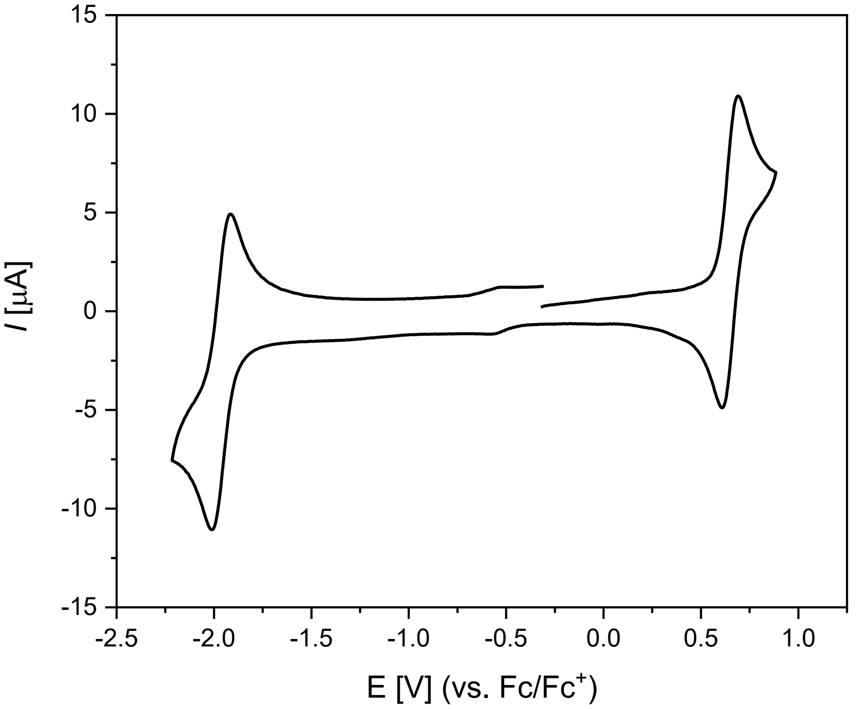

Figure S20: Cyclic voltammogram of **1-(Bpin)$_2$** in CH_2Cl_2 with [nBu$_4$N][PF$_6$] as the electrolyte and a scan rate of 250 mVs^{-1} referenced vs. the Fc/Fc$^+$ redox couple.

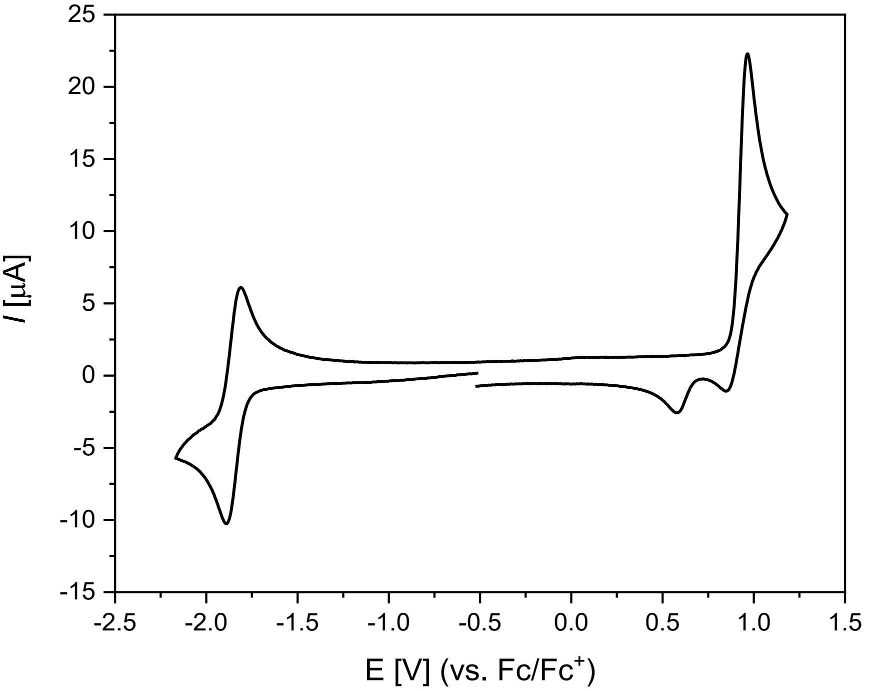

Figure S21: Cyclic voltammogram of **2-(Bpin)₂** in CH_2Cl_2 with [$n$$Bu_4N$][$PF_6$] as the electrolyte and a scan rate of 250 mVs^{-1} referenced vs. the Fc/Fc^+ redox couple.

TD-DFT Calculations

1

Calculated absorption spectrum

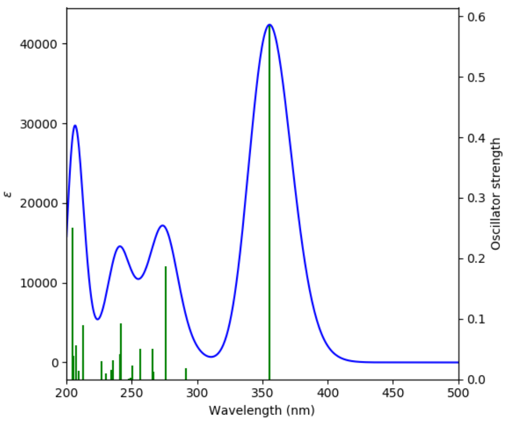

TD-DFT B3LYP/6-31+G(d), gas phase

Orbital	Energy [eV] Cam-B3LYP	Energy [eV] B3LYP
L+4	0.12	-1.10
L+3	-0.16	-1.38
L+2	-0.32	-1.54
L+1	-0.39	-1.59
LUMO	-1.19	-2.33
HOMO	-6.71	-5.49
H-1	-8.48	-7.06
H-2	-8.49	-7.09
H-3	-8.80	-7.36
H-4	-8.80	-7.38

Table S 2: Lowest energy singlet electronic transition of **1** (TD-DFT B3LYP/6-31+G(d), gas phase).

State	E [eV]	λ [nm]	*f*	Major contributions	Λ
**1**	3.49	355.54	0.5849	HOMO->LUMO (85%)	0.44
**2**	4.25	291.69	0.0178	HOMO->L+1 (14%), HOMO->L+5 (65%)	0.50
**3**	4.49	276.15	0.1867	HOMO->L+6 (91%)	0.58
**4**	4.65	266.55	0.0129	H-4->LUMO (13%), H-1->LUMO (30%), HOMO->L+5 (19%), HOMO->L+13 (13%)	0.44
**5**	4.67	265.69	0.0508	HOMO->L+4 (67%)	0.49
**6**	4.84	256.18	0.0497	H-1->LUMO (16%), HOMO->L+1 (57%), HOMO->L+3 (12%)	0.25
**7**	4.95	250.42	0.0232	HOMO->L+10 (40%), HOMO->L+11 (23%)	0.41
**8**	4.97	249.36	0.0027	HOMO->L+2 (74%)	0.18
**9**	5.01	247.56	0.0015	HOMO->L+3 (74%)	0.19
**10**	5.13	241.47	0.0927	H-6->LUMO (49%)	0.63
**11**	5.15	240.95	0.0412	H-8->LUMO (23%), H-7->L+1 (12%), H-6->LUMO (32%), H-6->L+2 (10%)	0.60
**12**	5.15	240.75	0.0044	H-9->LUMO (27%), H-8->L+3 (10%), H-7->L+2 (18%), H-6->L+1 (15%)	0.61
**13**	5.26	235.76	0.0315	H-7->LUMO (52%), H-3->LUMO (16%)	0.58
**14**	5.29	234.29	0.0153	HOMO->L+13 (33%)	0.43
**15**	5.39	230.23	0.0091	HOMO->L+7 (30%), HOMO->L+9 (38%)	0.28
**16**	5.46	227.10	0.0301	H-7->LUMO (33%), H-3->LUMO (26%), H-2->LUMO (21%)	0.51
**17**	5.61	220.92	0.0001	HOMO->L+12 (65%)	0.46
**18**	5.81	213.57	0.0000	HOMO->L+10 (35%), HOMO->L+11 (41%)	0.39
**19**	5.82	212.97	0.0309	H-4->LUMO (24%), H-2->L+6 (16%), HOMO->L+13 (10%)	0.53
**20**	5.83	212.57	0.0894	H-3->LUMO (16%), H-2->LUMO (32%), HOMO->L+11 (18%)	0.43
**21**	5.91	209.66	0.0147	HOMO->L+7 (11%), HOMO->L+9 (22%), HOMO->L+15 (46%)	0.29
**22**	5.93	209.01	0.0002	HOMO->L+8 (11%), HOMO->L+14 (31%), HOMO->L+17 (23%), HOMO->L+18 (15%)	0.29
**23**	5.98	207.21	0.0555	H-11->LUMO (13%), H-8->LUMO (19%), H-7->L+1 (15%)	0.63
**24**	6.03	205.49	0.0382	H-5->LUMO (20%), H-4->LUMO (23%), H-2->L+6 (11%)	0.41
**25**	6.05	204.83	0.2513	H-9->LUMO (36%), H-6->L+1 (12%), H-1->L+1 (19%)	0.52

Orbitals relevant to the $S_1 \leftarrow S_o$

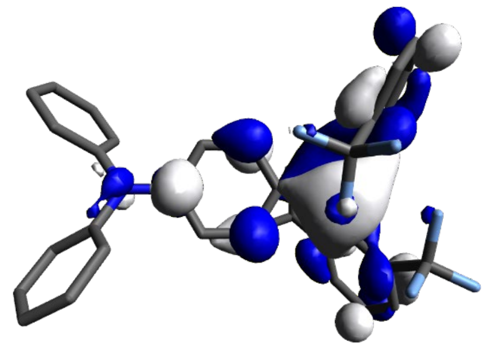

LUMO: –1.19 eV

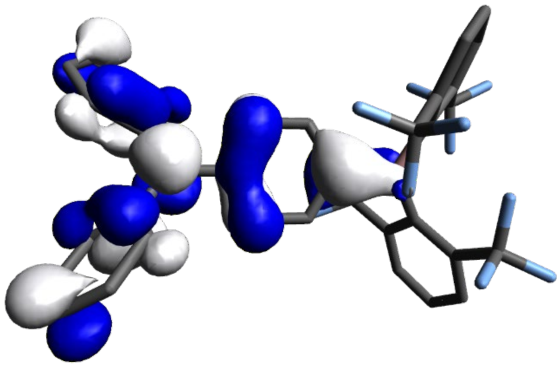

HOMO: – 6.71eV

Theoretical calculations: Cartesian coordinates

Compound 1

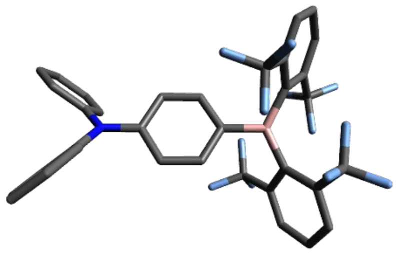

Point group: C_1

Total energy: – 1,622,547.83 kcal mol^{-1}

Dipole moment: 2.35 D

Imaginary frequencies: 0

C	2.05260400	0.97330700	-0.72247900
C	0.66742500	0.95301900	-0.73072600
C	-0.07592200	-0.00001800	0.00000300
C	0.66742700	-0.95300100	0.73081300
C	2.05260700	-0.97319600	0.72269300
C	2.77042800	0.00007000	0.00012200
N	4.17267200	0.00010100	0.00016900
B	-1.61903400	-0.00013400	-0.00006600
C	-2.33191400	-0.66952600	1.26750500
C	-2.33206100	0.66929600	-1.26751300
C	4.89691900	1.22123200	-0.09252900
C	4.89696700	-1.22100300	0.09287200
C	5.99789800	-1.32163700	0.95416400
C	6.71930600	-2.51306300	1.02923800
C	6.34359800	-3.61802900	0.25980200
C	5.24245900	-3.51740500	-0.59601200
C	4.52662600	-2.32427500	-0.68900100
C	5.99788800	1.32190000	-0.95376900
C	6.71924300	2.51335900	-1.02883100
C	6.34344700	3.61832300	-0.25943500
C	5.24226900	3.51766600	0.59632500
C	4.52648700	2.32450400	0.68930200
C	-3.19786700	-1.79093800	1.24526200
C	-3.63037800	-2.42318600	2.41877600
C	-3.24976200	-1.94732100	3.66546300
C	-2.45359400	-0.81107600	3.73245800
C	-2.00848400	-0.18757300	2.56436500
C	-3.19847100	1.79037700	-1.24507200
C	-3.63079400	2.42300700	-2.41846200
C	-3.24954300	1.94789100	-3.66523300
C	-2.45301700	0.81192000	-3.73246500
C	-2.00813100	0.18799300	-2.56450600
C	-1.18607500	1.06224100	2.81727300
F	-1.12132800	1.90699800	1.76735600
F	0.09209300	0.77092700	3.17880400
F	-1.70765900	1.78220900	3.84823300
C	-3.68584600	-2.45892100	-0.02321800
F	-2.85551200	-3.45971300	-0.41665100
F	-3.82534900	-1.61625300	-1.06880800
F	-4.90592800	-3.03297100	0.15772000
C	-1.18548600	-1.06159000	-2.81793000
F	-1.12082400	-1.90698900	-1.76855800
F	0.09269600	-0.76987900	-3.17909900
F	-1.70683300	-1.78098300	-3.84940900
C	-3.68730500	2.45775700	0.02340700
F	-2.85781100	3.45909200	0.41724200
F	-3.82652300	1.61485300	1.06882900
F	-4.90776400	3.03092500	-0.15784700
H	2.59170000	1.72565900	-1.28798200
H	0.14341800	1.70203100	-1.31777900
H	0.14342900	-1.70201400	1.31786900
H	2.59171300	-1.72548900	1.28826500
H	6.28316300	-0.46434900	1.55588200
H	7.57127700	-2.58015700	1.70044500
H	6.90357500	-4.54651100	0.32468100
H	4.94569100	-4.36653300	-1.20570400
H	3.67895600	-2.24052300	-1.36204600
H	6.28322300	0.46461500	-1.55545700
H	7.57124500	2.58047800	-1.69999700
H	6.90338400	4.54683000	-0.32430500
H	4.94543200	4.36679200	1.20598600
H	3.67878700	2.24072500	1.36230600
H	-4.27561200	-3.29114700	2.34797900
H	-3.58699600	-2.44088700	4.57137200
H	-2.18050900	-0.39587800	4.69658100
H	-4.27638200	3.29069300	-2.34749500
H	-3.58656500	2.44179300	-4.57103700
H	-2.17946000	0.39726800	-4.69669000

References

[1] R. Uson, L. A. Oro, J. A. Cabeza, H. E. Bryndza, M. P. Stepro, *Inorg. Synth.* **1985**, *23*, 126-130.

[2] F. Rauch, S. Fuchs, A. Friedrich, D. Sieh, I. Krummenacher, H. Braunschweig, M. Finze, T. B. Marder, *Chem. Eur. J.* **2020**, 10.1002/chem.201905559.

[3] A. Narsaria, F. Rauch, J. Krebs, P. Endres, A. Friedrich, I. Krummenacher, H. Braunschweig, M. Finze, J. Nitsch, F. M. Bickelhaupt, T. B. Marder, *Adv. Funct. Mater.* **2020**, accepted for publication March 2020.

[4] G. R. Fulmer, A. J. M. Miller, N. H. Sherden, H. E. Gottlieb, A. Nudelman, B. M. Stoltz, J. E. Bercaw, K. I. Goldberg, *Organometallics* **2010**, *29*, 2176-2179.

[5] G. M. Sheldrick, *Acta Crystallogr A Found Adv* **2015**, *71*, 3-8.

[6] G. M. Sheldrick, *Acta Crystallogr A* **2008**, *64*, 112-122.

[7] C. B. Hübschle, G. M. Sheldrick, B. Dittrich, *J. Appl. Crystallogr.* **2011**, *44*, 1281-1284.

[8] Diamond, 4.6.0, K. Brandenburg, Crystal Impact H. Putz & K. Brandenburg GbR, Bonn (Germany), **2017**.

[9] O. V. Dolomanov, L. J. Bourhis, R. J. Gildea, J. A. K. Howard, H. Puschmann, *J. Appl. Crystallogr.* **2009**, *42*, 339-341.

[10] Gaussian 09 Revision E.01, M. J. Frisch, G. W. Trucks, H. B. Schlegel, G. E. Scuseria, M. A. Robb, J. R. Cheeseman, G. Scalmani, V. Barone, B. Mennucci, G. A. Petersson, H. Nakatsuji, M. Caricato, X. Li, H. P. Hratchian, A. F. Izmaylov, J. Bloino, G. Zheng, J. L. Sonnenberg, M. Hada, M. Ehara, K. Toyota, R. Fukuda, J. Hasegawa, M. Ishida, T. Nakajima, Y. Honda, O. Kitao, H. Nakai, T. Vreven, J. A. Montgomery, J. E. Peralta, F. Ogliaro, M. Bearpark, J. J. Heyd, E. Brothers, K. N. Kudin, V. N. Staroverov, R. Kobayashi, J. Normand, K. Raghavachari, A. Rendell, J. C. Burant, S. S. Iyengar, J. Tomasi, M. Cossi, N. Rega, J. M. Millam, M. Klene, J. E. Knox, J. B. Cross, V. Bakken, C. Adamo, J. Jaramillo, R. Gomperts, R. E. Stratmann, O. Yazyev, A. J. Austin, R. Cammi, C. Pomelli, J. W. Ochterski, R. L. Martin, K. Morokuma, V. G. Zakrzewski, G. A. Voth, P. Salvador, J. J. Dannenberg, S. Dapprich, A. D. Daniels, Farkas, J. B. Foresman, J. V. Ortiz, J. Cioslowski, D. J. Fox, Gaussian Inc., Wallingford CT, **2016**.

[11] M. D. Hanwell, D. E. Curtis, D. C. Lonie, T. Vandermeersch, E. Zurek, G. R. Hutchison, *J. Cheminformatics* **2012**, *4*, 17.

[12] T. Lu, F. Chen, *J. Comput. Chem.* **2012**, *33*, 580-592.

[13] C. Lee, W. Yang, R. G. Parr, *Phys. Rev. B: Condens. Matter Mater. Phys.* **1988**, *37*, 785-789.

[14] G. A. Petersson, M. A. Al-Laham, *J. Chem. Phys.* **1991**, *94*, 6081-6090.

[15] G. A. Petersson, A. Bennett, T. G. Tensfeldt, M. A. Al-Laham, W. A. Shirley, J. Mantzaris, *J. Chem. Phys.* **1988**, *89*, 2193-2218.

[16] S. Grimme, *Chem. Eur. J.* **2004**, *10*, 3423-3429.

[17] T. Yanai, D. P. Tew, N. C. Handy, *Chem. Phys. Lett.* **2004**, *393*, 51-57.

[18] M. J. G. Peach, P. Benfield, T. Helgaker, D. J. Tozer, *J. Chem. Phys.* **2008**, *128*, 044118.
